# Latent likelihood ratio tests for assessing spatial kernels in epidemic models

**DOI:** 10.1007/s00285-020-01529-3

**Published:** 2020-09-05

**Authors:** David Thong, George Streftaris, Gavin J. Gibson

**Affiliations:** Maxwell Institute for Mathematical Sciences, Heriot-Watt University, Riccarton, Edinburgh EH14 4AS UK

**Keywords:** Spatio-temporal epidemic models, Bayesian inference, Latent likelihood ratio tests, Latent processes, 62F15, 92-08, 92D30, 62M30

## Abstract

One of the most important issues in the critical assessment of spatio-temporal stochastic models for epidemics is the selection of the transmission kernel used to represent the relationship between infectious challenge and spatial separation of infected and susceptible hosts. As the design of control strategies is often based on an assessment of the distance over which transmission can realistically occur and estimation of this distance is very sensitive to the choice of kernel function, it is important that models used to inform control strategies can be scrutinised in the light of observation in order to elicit possible evidence against the selected kernel function. While a range of approaches to model criticism is in existence, the field remains one in which the need for further research is recognised. In this paper, building on earlier contributions by the authors, we introduce a new approach to assessing the validity of spatial kernels—the latent likelihood ratio tests—which use likelihood-based discrepancy variables that can be used to compare the fit of competing models, and compare the capacity of this approach to detect model mis-specification with that of tests based on the use of infection-link residuals. We demonstrate that the new approach can be used to formulate tests with greater power than infection-link residuals to detect kernel mis-specification particularly when the degree of mis-specification is modest. This new tests avoid the use of a fully Bayesian approach which may introduce undesirable complications related to computational complexity and prior sensitivity.

**Electronic supplementary material:**

The online version of this article (10.1007/s00285-020-01529-3) contains supplementary material, which is available to authorized users.

## Introduction

Selection of spatial kernel functions in spatio-temporal epidemic models is a question of paramount practical importance. It is recognised (Shaw and Royle 1993; Gibson and Austin 1996) that predictions regarding the speed of epidemic spread or propensity for transmission over long distances are very sensitive to the choice of spatial kernel function. The control of epidemics such as foot and mouth disease (FMD) in the UK (Keeling 2001; Bates 2016; Chis Ster et al. 2009; Ferguson 2001; Ferguson et al. 2001; Jewell et al. 2009; Morris et al. 2001; BBC News 2011; Ster and Ferguson 2007; Streftaris and Gibson 2004a; Tildesley et al. 2008) or citrus canker in the USA (Neri et al. 2014; Gottwald et al. 2002a, b) has proved controversial on account of the removal of healthy hosts as part of the strategy. Such strategies have been informed by mathematical models in which the choice of spatial kernel has been a factor in determining a ‘culling radius’ (for example Keeling 2001; Ferguson 2001). Methods for model criticism and comparison are therefore much-needed to ensure that, as far as possible, such decisions can be supported and defended in the light of available evidence.

Although several approaches to model criticism for epidemic models exist, in the epidemic context many of these suffer from certain difficulties which motivate the development of further approaches. In Gibson et al. (2018) the approaches commonly used are reviewed. These range from Bayes factors and Bayesian model selection, posterior predictive *p* values and latent classical tests, and the use of the deviance information criterion (DIC) (Spiegelhalter et al. 2002, 2014) including missing data variants (Celeux et al. 2006). To summarise some findings from the paper: issues with model assessment for stochastic spatio-temporal epidemic models essentially arise primarily because exact infection times cannot be directly observed (due to latent period of the infection, delay in reporting etc.). These infection times are governed by the spatial transmission kernel, which is often the aspect of model fit with which modellers are primarily concerned.

As a result, Bayes factors and Bayes model selection suffer from prior sensitivity, as well as difficulty in devising appropriate algorithms. In addition, the DIC has an infinite number of possibilities in the missing data scenario (Celeux et al. 2006), each all yielding model rankings that differ from each other. The latent classical testing approach does not suffer from these drawbacks.

One recommendation from Gibson et al. (2018) is that it is prudent to follow the advice of Box (1976) that one should test selectively for those forms of mis-specification which are most strongly suspected and to design specific tests for this purpose. This is the approach that is taken throughout this paper where we will formulate latent likelihood ratio-tests (Streftaris and Gibson 2004a, 2012) for kernel mis-specification and compare their sensitivity with that of the infection-link residuals test introduced in Lau et al. (2014). Both of these methods lie within the field of posterior predictive checking and latent classical testing approaches (Meng 1994; Gelman 2013; Gelman et al. 1996) which fuse Bayesian and classical thinking by having a Bayesian observer impute the result of a classical goodness-of-fit test applied to a latent process, where the process and the test can be specified flexibly to maximise the chance of detecting the suspected mis-specification, should it be present. The approach differs from a purely Bayesian one, in which modes of mis-specification are accommodated through the process of Bayesian model expansion Draper (1995), the representation of several competing models within an expanded model where each model has an index. Bayesian reasoning is then used to compare models via the posterior distribution of the model index. One reason for not adopting this latter approach is that inference for relatively simple epidemic models using partial observation is already a complex process. We therefore seek model comparison methods that can be utilised without increasing the dimension of the models to which Bayesian methods are applied. Accordingly, the methods we present can be integrated into analyses without increasing the complexity of the fundamental Bayesian computations.

We will consider stochastic models for an infectious disease spreading through a closed population of spatially-distributed hosts exemplified by the spatio-temporal Susceptible-Exposed-Infectious-Removed (SEIR) model. It will be assumed that the locations of hosts are known and fixed. Under this model, the host population at time *t* is partitioned into subsets *S*(*t*), *E*(*t*), *I*(*t*) and *R*(*t*). Hosts in *S*(.) are susceptible to infection, hosts in *E*(.) have been infected but are not yet able to transmit, hosts in *I*(.) can pass on infection, while hosts in *R*(*t*) have been removed (e.g. by death, hospitalisation, or the acquisition of immunity) and play no further part in the epidemic. A susceptible individual at coordinates $\mathbf{x}$ at time *t* becomes exposed with probability:

1 $$\begin{aligned} \mathrm {Pr}(\text {Individual becomes exposed during }[t,t+dt]) =C(t)\;dt+o(dt) \end{aligned}$$

where,

2 $$\begin{aligned} C(t) = \alpha + \beta \sum _{\mathbf{y} \in I(t)} K(\kappa , \mathbf{x}, \mathbf{y}) \end{aligned}$$

where *I*(*t*) comprises sites infectious at time *t*, $\alpha$ and $\beta$ are *primary* and *secondary* infection rates, and $\kappa$ parametrises the *spatial kernel function* *K*(). For convenience, we identify hosts with their location. The choice of *K* greatly influences the design of control strategies, for example based on ring-culling. A longer-tailed kernel may suggest the use of a larger culling radius and vice versa. Sojourn times in the *E* and *I* class are modelled using appropriate distributions such as Gamma or Weibull distributions. We will denote by $\theta$ the vector of model parameters formed from $\alpha$, $\beta$, $\kappa$ supplemented by parameters specifying the distributions of sojourn times in *E* and *I*. This flexible framework can accommodate complexity arising e.g. from host heterogeneity (differences between hosts that affect their susceptibility and/or infectivity) as appropriate (Parry et al. 2014; Jewell et al. 2009).

When data *y* contain partial information (e.g. removals or ‘snapshots’ of *I*(*t*) at discrete times using imperfect diagnostic tests) data-augmented Bayesian analysis is now a standard tool for investigating $\pi (\theta |y)$ via $\pi (\theta , z |y)$, where *z* incorporates unobserved transitions and, possibly, graphs of infectious contacts. Computations are often effected using reversible-jump MCMC or particle filtering (King et al. 2008). In this paper, we will assume that observations include times and locations of all transitions from *E* to *I* and from *I* to *R*, so that the subsets *I*(*t*) and *R*(*t*) are observed but individuals in *S*(*t*) cannot be distinguished from those in *E*(*t*). Such data which can be modelled by an SEIR model are encountered in many real-world situations, for example, concerning diseases with long latent periods or because of delay in reporting of cases, such as Foot-and-Mouth Disease (Keeling 2001; Jewell et al. 2009; Chis Ster et al. 2009; Ferguson 2001; Ferguson et al. 2001; Jewell et al. 2009; Morris et al. 2001; Ster and Ferguson 2007; Streftaris and Gibson 2004a; Tildesley et al. 2008) and Citrus Canker (Neri et al. 2014; Gottwald et al. 2002a, b). In many such datasets, the situation does arise where the times of infectiousness and removal are observed, but not the time of exposure, for example, in diseases where infectivity occurs only after the onset of symptoms, for example smallpox and avian influenza (Rorres et al. 2011; Stockdale et al. 2017; Boys and Giles 2007), or in insect or plant infestations (Brown et al. 2013; Lau et al. 2014), where the invading species has to reach a certain phase of its life cycle before producing eggs/seeds/spores. We therefore specify *z* to incorporate the times and location of the unobserved transitions from *S* to *E* (termed *exposure* events) and use MCMC to sample from $\pi (\theta , z |y)$. As the number of exposure events is not uniquely determined by the data, the state-space for $(\theta , z)$ comprises components of varying dimension requiring the use of reversible-jump methods. It is straightforward to apply the methods used on this class of models, to snapshot data or other forms of partial observation.

The rest of the paper is organised as follows. In Sect. 2, we discuss the general features of the posterior-predictive, latent classical testing framework before describing how functional-model representations of epidemic models have been used in the specification of *infection-link residuals (ILR)*Lau et al. (2014). In Sect. 3, we explicitly formulate new latent classical tests for detecting kernel mis-specification using likelihood ratios, where the ratio is based on a complete or partial parameter likelihood. In Sect. 4, we apply the tests to simulated data comparing the ability or ‘power’ of the likelihood-based and infection-link residual tests to detect kernel mis-specification in several scenarios. Conclusions are summarised in Sect. 5.

## Posterior predictive checks and latent classical tests

The model assessment methods utilised here have their roots in the calibrated Bayesian approach promoted by several authors in which parameter estimation is achieved using Bayesian reasoning and model assessment draws from classical approaches applied to posterior predictive distributions of outcomes (Guttman 1967; Rubin 1981, 1984). Throughout we consider the situation where a Bayesian observer *B* observes the outcome *y* of an experiment with proposed statistical model $\pi _0(y | \theta )$ where beliefs regarding the parameter vector $\theta$ are represented by the prior distribution $\pi _0(\theta )$. We suppose that the likelihood $\pi _0(y | \theta )$ may not necessarily be tractable—a situation which typically applies in the case of a partially observed epidemics and which complicates investigation of the posterior density $\pi _0(\theta | y)$.

Meng (1994) proposed the use of posterior predictive *P*-values as a means of model assessment, this being the posterior probability that some test statistic $T(y^{rep})$ from a replicate experiment, yielding data $y^{rep}$, independent (given $\theta$) of the one observed, exceeds the observed value *T*(*y*). Mathematically, this Bayesian *P*-value is given by the integral

$$\begin{aligned} p(y)&=P(T(y^{rep})>T(y)|y)\\&=\int P\left( T(y^{rep})>T(y)|\theta \right) \pi _0(\theta |y)d\theta \end{aligned}$$

The idea is extended in Meng (1994), Gelman et al. (1996) to include test statistics *T* that are functions of the parameters, known as *discrepancy variables*, to give the more general formulation

$$\begin{aligned} p(y)=\int P\left( T(y^{rep},\theta )>T(y,\theta )|\theta \right) \pi _0(\theta |y)dx\,d\theta \end{aligned}$$

The methods that we propose are based on these ideas. We highlight three main features of the approach as applied here.

- Writing $\Pr \left( T(y^{rep},\theta )>T(y,\theta )|\theta \right) = p(y, \theta )$, we can write $p(y) = {\mathbb {E}}\left( p(y, \theta ))|y\right)$. In the case where $T(y, \theta )$ follows a continuous distribution with respect to *y* the prior distribution of $p(y, \theta )$ is distributed as *U*(0, 1). The laws of conditional variance force the prior distribution of *p*(*y*) under the assumed model to be typically less variable that a *U*(0, 1) (Meng 1994). Therefore, we tend to view $p(y, \theta )$ as the *P*-value of interest, as its prior distribution matches the sampling distribution of a *P*-value in the classical setting, with the usual *p*(*y*) being its posterior expectation. In this framework, evidence against the model may be expressed as the posterior probability that $p(y, \theta )$ falls below some value; of course, the posterior expectation of $p(y, \theta )$ provides a further natural summary measure that we utilise.
- We make use of discrepency variables that are functions of latent processes. Specifically let *r* be a process varying jointly with *y* according to the model $\pi (y, r | \theta )$ for which the marginal model $\pi (y|\theta )$ coincides with $\pi _0(y | \theta )$. Then we can assess the model $\pi _0(y | \theta )$ by considering the posterior distribution of *P*-value based on a discrepency variable $T(r, \theta )$ or $T(r, y, \theta )$. As discussed in Gibson et al. (2018), in this framework we can consider the Bayesian *B* to be imputing the *P*-value from test of the model $\pi (r | \theta )$ carried out by a classical observer *C* with knowledge of *r* and $\theta$. The above approach is natural in the setting of epidemic modelling, discussed in the previous section, where data-augmentation may require unobserved exposure events to be imputed when sampling from $\pi (\theta , z |y)$. One issue that arises with the use of latent, imputed processes in this way is that the result of the latent test may be dominated by the data imputed using the model—particularly if imputation is extensive. See Gelman (2013) for an example of this phenomenon involving the Normal distribution and Streftaris and Gibson (2012) for an example in the epidemic setting. While it may be attractive to base discrepency variables on latent processes in order to specify tests that may be analytically tractable, or powerful in the classical sense as in the case of the likelihood-ratio tests considered in the next section, one should be mindful of the tendency of the imputed data to reinforce the model being tested.
- Suppose that $\pi _j(y, r_j |\theta ), j = 1, ..., k$ represent models for the joint distribution of $(y, r_j)$ all of which specify the same *marginal* model $\pi _0(y|\theta )$ and share a common parameter prior distribution $\pi (\theta )$. Observation of *y* alone carries no information on the relative validity of these models. That is, *y* carries exactly the same evidence *against* every model with marginal $\pi _0(y | \theta )$. Therefore, if we wish, we can design the latent process *r* to yield a test that is particularly sensitive to a suspected form of model mis-specification. This facility is exploited in the case of infection-link residuals discussed later.

When the latent process *r* has a fixed distribution that does not depend on $\theta$ we may consider *r* to play the role of a residual process and the latent *P*-value can be obtained via a test of the fit of the imputed *r* to this process. In Gibson et al. (2006) epidemic models were tested using this approach where *r* was a set of imputed Sellke thresholds, distributed as independent draws from $\mathrm{Exp}(1)$ under the assumed model. The infection-link residuals, used to test spatial kernels in Lau et al. (2014), and considered further in this paper provide a further example.

### Infection-link residuals

The starting point is to construct a functional-model representation of the epidemic process. In this formalism the observations *y* are represented as a deterministic function $x = h(r, \theta )$ of $\theta$ and some unobserved process *r* with fixed distribution independent of $\theta$, where $x=(y,z)$. This means that *r* can be treated as a residual process and tests for compliance with the specified distribution can be applied to the imputed realisations of *r*. Such an approach fits well for epidemic models where sampling from $\pi (\theta , r | y)$ is often possible using Markov chain Monte Carlo methods.

In Lau et al. (2014) a functional-model for a spatio-temporal SEIR model is presented where the process *r* is composed of four independent i.i.d. Unif(0, 1) sequences, $r_1, r_2, r_3, r_4$. Consider the mapping $x = h_\theta (r_1, r_2, r_3, r_4)$, where *x* records the time and nature of every event occurring during the epidemic. Details can be found in Lau et al. (2014). The time of each subsequent infection event is determined from the process $r_1 = \{ r_{1j}, j \ge 1\}$ while processes $r_3$ and $r_4$ specify the quantiles of the sojourn periods in the *E* and *I* class respectively for each infection. The infection-link residual sequence (to which tests are applied) $r_2 = \{r_{2j}, j \ge 1 \}$ determine the particular *I*-*S* pair responsible for each infection event. Given the time of the $j^{th}$ infection, $t_j$, we identify the set of *I*-*S* links

$$\begin{aligned} S = \{ K( \mathbf{x}, \mathbf{y}, \kappa ) | \mathbf{x} \in S(t_j), \mathbf{y} \in I(t_j) \} \end{aligned}$$

and order these according to ascending order of magnitude of $K( \mathbf{x}, \mathbf{y}, \kappa )$. The particular link causing the $j^{th}$ infection is selected by considering the cumulative sum of the ordered links and identifying the first link where this cumulative sum exceeds the value $r_{2j}W$ where *W* denotes the sum of the weights in *S*. It is straightforward to explore the joint posterior $\pi (\theta , r_1, r_2, r_3, r_4 | y)$. If the kernel function *K* has been misspecified (for example by underestimating the propensity for long-range transmission by assuming an exponentially bounded form when a power-law relation is more appropriate), then when the process $r_2$ is imputed, some systematic deviation from a *U*(0, 1) should be anticipated. In Lau et al. (2014) p-values were imputed from an Anderson-Darling test (Anderson and Darling 1954) applied to $r_2$ and it was demonstrated that the approach can detect kernel mis-specification in simulated data sets. In this paper we investigate whether it is possible to improve on the sensitivity of the ILR tests using likelihood-based methods.

## Latent likelihood ratio tests for model comparison

The general approach of embedding likelihood ratio tests has been followed previously in the epidemic setting in Streftaris and Gibson (2004a) where results of an ANOVA test applied to viraemic measurements taken on a host population, partitioned by depth in an unobserved infection graph, were imputed. It is also used in Streftaris and Gibson (2012) to compare threshold models for tolerance to infection. While the ILR test is targeted at generic forms of model inadequacy (mis-specification of the tail properties of a spatial kernel), latent likelihood ratio tests demand that a specific alternative model is identified and therefore provide a means of model comparison.

To test model $M_0$ with likelihood $\pi _0(y|\theta )$ against a model $M_1$ with parameter $\theta _1$ and likelihood $\pi _1(y)$, it is natural for *B* (the Bayesian observer) to impute $C's$ (the classical observer’s) conclusion from a generalised *likelihood ratio test* (LRT) using the discrepancy variable $T(y, \theta ) = \frac{\pi _0(y | \theta )}{\pi _1(y | {{\hat{\theta }}}_1)}$ where ${{\hat{\theta }}}_1$ is the *maximum likelihood estimate* (MLE) of the parameter $\theta _1$ in $M_1$, calculated using the imputed augmented data. For epidemic models and data, $\pi (y |\theta )$ and $\pi _1(y|\theta _1)$ would typically be intractable. Nevertheless, *B* can impute $(\theta , z)$, where *z* represents an appropriate latent process, and the conclusion of $C's$ test based on the generalised likelihood ratio $T(x, \theta ) = \frac{\pi _0(x | \theta )}{\pi _1(x|{{\hat{\theta }}}_1)}$, where $x=(y,z)$, so long as $\pi _0(x | \theta )$ and $\pi _1(x | \theta _1)$ are tractable. Note that $T(x, \theta )$ is the ratio of likelihoods as calculated from augmented data, not the observed data. We will therefore refer to $\pi _0(x | \theta )$ and $\pi _1(x | \theta )$ as the *augmented data likelihood*. This can be interpreted as the likelihood that would be calculated by an observer of the augmented data set.

Suppose that, given partial information *y*, we use data-augmented MCMC to explore $\pi _0(\theta , x |y)$, where *x* comprises the times and nature of all transitions within an observation window $(0, T_{max})$. The latent likelihood ratio test may be implemented as an addendum to this analysis as follows.

1. B draws samples $(\theta , x)$ from $\pi _0(\theta , x | y)$.
2. For each sample $(\theta ^{(i)}, x^{(i)})$ B proceeds to:
  calculate the maximum likelihood estimate (MLE), $\hat{\theta }_1^{(i)}$, of the parameter $\theta _1$, under the alternative model,
  compute the ratio $T(x^{(i)}, \theta ^{(i)}) = \frac{\pi _0(x^{(i)}|\theta ^{(i)})}{\pi _1(x^{(i)} | \hat{\theta }_1^{(i)})}$ and the associated *P*-value
    $$\begin{aligned} p(\theta ^{(i)}, x^{(i)}) = \Pr (T(x, \theta ^{(i)}) < T(x^{(i)}, \theta ^{(i)}) | \theta ^{(i)},x^{(i)}), \end{aligned}$$
    where *x* is drawn randomly from $\pi _0(x | \theta ^{(i)})$.

By repeating these steps within a standard MCMC analysis, a sample from $\pi (p(\theta , x) | y)$ can be obtained.

We do not assume nesting of models that might allow asymptotic results on sampling distributions of likelihood ratios to be applied. For each sampled pair $(\theta ^{(i)}, x^{(i)})$ we may estimate the *P*-value by simulation. The simplest approach is to estimate the posterior expectation of the *P*-values as follows:

- Compute the ratio $T(x^{(i)}, \theta ^{(i)}) = \frac{\pi _0(x^{(i)}|\theta ^{(i)})}{\pi _1(x^{(i)} | \hat{\theta }_1^{(i)})}$. Simulate a random draw, $x'$ from $\pi _0(x|\theta ^{(i)})$, obtain the MLE, ${{\hat{\theta }}}'_1$, by maximising $\pi _1(x'|\theta _1)$, and compute $T' = \frac{\pi _0(x'|\theta )}{\pi _1(x' |{{\hat{\theta }}}'_1)}$.

An estimate of the posterior mean of $\pi _0(p(\theta , x) | y)$, is obtained from the frequency with which $T' < T(x^{(i)}, \theta ^{(i)})$. This quantity provides some information on the strength of the evidence against the modelling assumptions. To investigate the full posterior distribution of $p(\theta , x)$ we can draw multiple independent $x'$ for each $\theta ^{(i)}$ and compute the proportion of these for which $T' < T(x^{(i)}, \theta ^{(i)})$.

A major motivating factor for considering the latent process *x* is that $\pi (x | \theta )$ is analytically tractable and a likelihood ratio test (generally agreed to be a powerful approach to model comparison) can be implemented. This avoids the need to approximate the observed data likelihood $\pi (y | \theta )$, for example using synthetic likelihoods (for example, Wood 2010) or ideas from Approximate Bayesian Computation (for example, McKinley et al. 2009; Csilléry et al. 2010; Sisson et al. 2018) (see discussion). At the same time, the approach of using the imputed *x* may suffer from the issue of reinforcement discussed earlier since *x* is imputed by conditioning on the model being tested. In the appendix, we explore this phenomenon in the case of likelihood ratio tests and the desirability of imputing as little information as possible beyond the observations, *y*. For this reason, we will explore two forms of latent likelihood ratio test which differ in terms of the amount of imputed information utilised.

### Latent likelihood tests for kernel assessment

We now consider the situation where $M_0$ and $M_1$ denote epidemic models of the same general form as described in Eq. 1 which differ only in the choice of spatial kernel function $K(d, \kappa )$. Bayesian *B* proposes an SEIR model for an emerging epidemic of the form described in Sect. 1. The model, $M_0$, incorporates a transmission kernel $K_0(d, \kappa _0)$ and a prior $\pi _0(\theta )$ is assigned to the parameter vector $\theta _0 = (\alpha , \beta , \kappa _0, \theta _E, \theta _I)$. We consider two forms of latent likelihood test for kernel comparison.

#### Full-trajectory latent likelihood ratio test (LLRT)

This analysis is achieved through *B* investigating $\pi _0(\theta _0, x | y)$, where *x* is the complete trajectory of the epidemic (the times of all exposure events and locations of the exposure, infection and removal events not considering the infection tree). The MCMC algorithm used to do this is standard (for example, Gibson and Renshaw 1998; O’Neill and Roberts 1999; Streftaris and Gibson 2004a, b; Forrester et al. 2006; Gibson et al. 2006; Chis Ster et al. 2009; Starr et al. 2009; Neri et al. 2014) and is summarised in Electronic Supplementary Material Appendix 1. For each sample $(\theta _0, x)$, the MLE ${{\hat{\theta }}}_1$ is computed using the optimisation routine described in Electronic Supplementary Material Appendix 2, and the algorithm is implemented as in Sect. 3. The test statistic used is the augmented data likelihood ratio, as detailed in Step 2 in Sect. 3.

#### Partial LLRT

In this setting, Observer *B* investigates $\pi _0(\theta _0, x | y)$ but Observer *C* is provided only with $\theta _0$ and *z*, where *z* incorporates for each exposure event, *j*:

- the sets of locations of susceptible and infectious individuals, $S(t_j-)$, $I(t_j-)$ immediately prior to the time of the event, $t_j$;
- the location of the exposed individual, $\mathbf{x}_j \in S(t_j-)$.

The times or even the order of the exposure events are not included in *z* though some restrictions on the latter will follow from *z*. Let $G_0(\theta _0, z)$ be defined by

$$\begin{aligned} G_0(\theta _0, z) = \prod _j \frac{\alpha + \beta \sum _{\mathbf{} y \in I(t_j-)} K_0(|\mathbf{y} - \mathbf{x}_j|, \kappa _0)}{|S(t_j-)|\alpha + \beta \sum _{\mathbf{y} \in I(t_j-), \mathbf{x} \in S(t_j-)} K_0(|\mathbf{y} - \mathbf{x}|, \kappa _0)} \end{aligned}$$

where $|S(t_j-)|$ denotes the cardinality of $S(t_j-)$. An analogous partial likelihood for $M_1$ with kernel function $K_1$ and parameter $\theta _1$ is given by

$$\begin{aligned} G_1(\theta _1, z) = \prod _j \frac{\alpha + \beta \sum _{\mathbf{} y \in I(t_j-)} K_1(|\mathbf{y} - \mathbf{x}_j|, \kappa _1)}{|S(t_j-)|\alpha + \beta \sum _{\mathbf{y} \in I(t_j-), \mathbf{x} \in S(t_j-)} K_1(|\mathbf{y} - \mathbf{x}|, \kappa _1)}. \end{aligned}$$

Then, if ${{\hat{\theta }}}_1$ maximises $G_1(\theta _1, z)$ we can define a partial likelihood ratio statistic

$$\begin{aligned} T_{partial}(\theta _0, z) = \frac{G_0(\theta _0, z)}{G_1({{\hat{\theta }}}_1, z)}. \end{aligned}$$

This statistic is used in place of the augmented data likelihood ratio in Step 2 in Sect. 3.

The partial LLRT requires that only $\theta _0$ and *z* are imputed by *B* for its calculation. Thus, the impact of reinforcement of $M_0$ may be lessened. Moreover, if detection of a possibly misspecified kernel is the goal, then $T_{partial}(\theta _0, z)$ is a statistic which ‘focuses’ on this aspect of the model. It is therefore possible that the partial LLRT, at least in some circumstances, may be more effective in eliciting evidence of a mis-specified kernel than the augmented data likelihood LLRT. In the Appendix, we explore the impact of reinforcement on the performance of the LLRT in terms of its power to detect a mis-specified model and how this may vary with the amount of information imputed. Moreover, the partial LLRT is a natural comparator for the ILR test used in Lau et al. (2014), as, for both of these tests, $(\theta _0, z)$ is necessary and sufficient for computation of the test result.

In the next section we consider the ability of the ILR, and the two LLRTs to detect mis-specification of the transmission kernel in a spatio-temporal epidemic model in a simulation study.

## Simulation study

In keeping with the assumptions of Lau et al. (2014), we assume that the observations *y* record the transitions from *E* to *I* and from *I* to *R*, but that exposure events are not recorded. Analogous data have been encountered in many real-world situations, for example in diseases where infectivity occurs only after the onset of symptoms, for example smallpox and avian influenza (Rorres et al. 2011; Stockdale et al. 2017; Boys and Giles 2007), or in insect or plant infestations (Brown et al. 2013; Lau et al. 2014), where the invading species has to reach a certain phase of its life cycle before producing eggs/seeds/spores which are laid on other hosts. Epidemics are simulated in an initially totally susceptible population of 1000 hosts uniformly distributed over a square region of size $2000\times 2000$ units. Both primary and secondary infection are present and an exponentially decaying spatial kernel function of the form

$$\begin{aligned} K( \kappa , {\mathbf {x}}, {\mathbf {y}}) = \exp (-\kappa |{\mathbf {x}}- {\mathbf {y}}|) \end{aligned}$$

is assumed, where ${\mathbf {x}}$ and ${\mathbf {y}}$ denote the positions of two hosts. We assume that the sojourn times in the *E* and *I* classes follow Gamma distributions with means and variances $\mu _E, \mu _I$ and $\sigma ^2_E, \sigma ^2_I$ respectively. Table 1 lists the parameter values used to simulate the data. These parameters are based on those used in the simulation study of the ILR test in Lau et al. (2014), to allow comparison with the simulation study therein. Starting from an entirely susceptible population, the epidemic is simulated until a fixed percentage of the population (100%, 40% or 70%) was observed as infected. Four different parameter sets are used—a baseline scenario, and the same parameter set with $\alpha$, $\beta$ and $\kappa$ respectively increased to twice the baseline value. The baseline set of parameter values, and the modified values, are given in Table 1.

**Table 1:** Table of the parameters used in the generation of the simulated data-sets used in Sect. 4

Parameter	Data-set			
	Original	$\alpha \times 2$	$\beta \times 2$	$\kappa \times 2$
$\alpha$	0.001	$\mathbf {0.002}$	0.001	0.001
$\beta$	3.000	3.000	$\mathbf {6.000}$	3.000
$\kappa$	0.030	0.030	0.030	$\mathbf {0.060}$
$\mu _{E}$	5.000	5.000	5.000	5.000
$\sigma _{E}^{2}$	2.500	2.500	2.500	2.500
$\mu _{I}$	1.772	1.772	1.772	1.772
$\sigma _{I}^{2}$	0.858	0.858	0.858	0.858

For each simulated epidemic, each test was applied using 3 different observation windows corresponding to the intervals up to which 100%, 70% or 40% of the population was observed to be infected. The likelihood-based tests as implemented only allow for estimation of the posterior expectation of an imputed p-value, and we therefore use the posterior expectation as the summary measure of evidence for all the tests (even though the full posterior can be explored for the ILR test).

To each simulated data set *y* we fit two separate misspecified models with isotropic kernel functions:

$$\begin{aligned} K( \kappa , \mathbf{x}, \mathbf{y} )= & {} (1 + |{\mathbf {x}}-{\mathbf {y}}|^\kappa )^{-1};\\ K( \kappa , \mathbf{x}, \mathbf{y})= & {} \exp (-\kappa |{\mathbf {x}}-{\mathbf {y}}|^2). \end{aligned}$$

In the former case, infective challenge decreases according to a power-law, while in the latter case the Gaussian kernel is exponentially bounded. Informally, we may consider the first kernel to represent a more severe degree of mis-specification, in comparison to the real exponential kernel, than the second one. We may anticipate that tests should find more evidence against the assumptions when the power-law kernel is fitted. The fitted model, whose adequacy is to be tested, will be referred to as $M_0$. The main distinction between spatial transmission kernels is whether or not they are exponentially bounded (Gibson and Austin 1996; Shaw 1995). In this paper, we have selected two kernels that deliberately misfit data generated from a known exponential kernel: a Gaussian kernel, and a long-tailed power law kernel. The Gaussian kernel is exponentially bounded, whilst the power-law kernel is not.

The simulated data are generated in all data-sets from an exponential kernel. This model is referred to as $M_1$, and will be the model that $M_0$ is compared against in the LLR tests. This exponential kernel is given by:

$$\begin{aligned} K( \kappa , \mathbf{x}, \mathbf{y})= & {} \exp (-\kappa |{\mathbf {x}}-{\mathbf {y}}|).\\ \end{aligned}$$

In all cases we use non-informative prior distributions for the parameters in the fitted model as follows: A $\mathrm {Unif}(0,M)$ uniform prior was used for $\alpha ,\mu _{E},\sigma _{E}^{2},\mu _{I},\sigma _{I}^{2}$, where $M\approx 1.7\times 10^{308}$ is the computer limit for double precision floating-point numbers in C++.

The prior distributions used for the other parameters were:

$$\begin{aligned} \beta&\sim \mathrm {Gamma}(\mu =1,\sigma ^{2}=100)\\ \kappa&\sim \mathrm {Gamma}(\mu =1,\sigma ^{2}=100) \end{aligned}$$

**Fig. 1:**
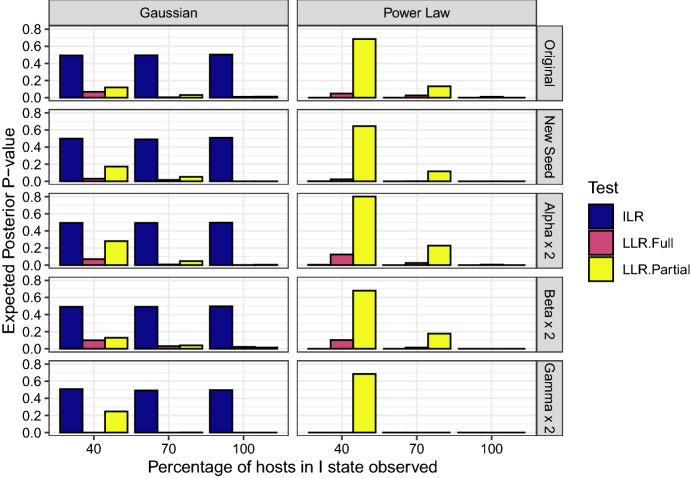
Comparison of Latent Likelihood Ratio (LLR) test to Infection Link Residuals test: Plot of a grid of bar-charts of the expected posterior predictive *p*-values for all simulated datasets generated. Each row contains the results for each dataset. “Original” is the dataset generated with the original base set of parameters. “New Seed” is the dataset generated with the original base set of parameters but with a different random number generator seed. “Alpha x 2”, ‘Beta‘ x 2” and “Gamma x 2” are the datasets with $\alpha$, $\beta$ or $\gamma$ twice that of the original parameter set. Each column shows the results for each kernel that was deliberately misfit to the data, which is generated from a known exponential kernel. “Gaussian” denotes a Gaussian kernel was fitted, and “Power Law” denotes that a power-law kernel was fitted

The results of the simulation study are presented in Table 2 and in Fig. 1. Some obvious trends can be seen.

**Table 2:** Comparison of Latent Likelihood Ratio (LLR) test to Infection Link Residuals test: data-set, $M_0$ tested and estimated expected *p*-values from the infection link residuals test, LLR (augmented data likelihood) and LLR (partial likelihood)

Data-set	$M_{0}$	Total % Infections Observed	ILR $\hat{E(p)}$	LLR (augmented data) $\hat{E(p)}$	LLR (Partial) $\hat{E(p)}$
$\alpha \times 2$	$\left( 1+d^{\kappa }\right) ^{-1}$	100	0.0000243	0.005319	0.0000000
$\alpha \times 2$	$\left( 1+d^{\kappa }\right) ^{-1}$	70	0.0002571	0.02473	0.2269000
$\alpha \times 2$	$\left( 1+d^{\kappa }\right) ^{-1}$	40	0.0042040	0.1242	0.8014000
$\alpha \times 2$	$\exp \left\{ -\kappa d^{2}\right\}$	100	0.4966585	0.0006974	0.0038500
$\alpha \times 2$	$\exp \left\{ -\kappa d^{2}\right\}$	70	0.4929907	0.006932	0.0461200
$\alpha \times 2$	$\exp \left\{ -\kappa d^{2}\right\}$	40	0.4937340	0.06909	0.2801000
$\beta \times 2$	$\left( 1+d^{\kappa }\right) ^{-1}$	100	0.0000006	0.0000	0.0000000
$\beta \times 2$	$\left( 1+d^{\kappa }\right) ^{-1}$	70	0.0000013	0.01566	0.1771000
$\beta \times 2$	$\left( 1+d^{\kappa }\right) ^{-1}$	40	0.0001413	0.1031	0.6801000
$\beta \times 2$	$\exp \left\{ -\kappa d^{2}\right\}$	100	0.4963660	0.02189	0.0157500
$\beta \times 2$	$\exp \left\{ -\kappa d^{2}\right\}$	70	0.4905312	0.03135	0.0393000
$\beta \times 2$	$\exp \left\{ -\kappa d^{2}\right\}$	40	0.4907014	0.1000	0.1295000
$\kappa \times 2$	$\left( 1+d^{\kappa }\right) ^{-1}$	100	0.0004014	0.0000000	0.0000000
$\kappa \times 2$	$\left( 1+d^{\kappa }\right) ^{-1}$	70	0.0002682	0.0000000	0.0000000
$\kappa \times 2$	$\left( 1+d^{\kappa }\right) ^{-1}$	40	0.0000569	0.0000000	0.6845000
$\kappa \times 2$	$\exp \left\{ -\kappa d^{2}\right\}$	100	0.4970400	0.0000000	0.0000000
$\kappa \times 2$	$\exp \left\{ -\kappa d^{2}\right\}$	70	0.4920980	0.0000000	0.0021380
$\kappa \times 2$	$\exp \left\{ -\kappa d^{2}\right\}$	40	0.5088031	0.0000000	0.2474000
Original	$\left( 1+d^{\kappa }\right) ^{-1}$	100	0.0000013	0.000000	0.0000000
Original	$\left( 1+d^{\kappa }\right) ^{-1}$	70	0.0000011	0.02533	0.1325000
Original	$\left( 1+d^{\kappa }\right) ^{-1}$	40	0.0000569	0.04713	0.6845000
Original	$\exp \left\{ -\kappa d^{2}\right\}$	100	0.5026800	0.009208	0.0108500
Original	$\exp \left\{ -\kappa d^{2}\right\}$	70	0.4943048	0.004225	0.0294100
Original	$\exp \left\{ -\kappa d^{2}\right\}$	40	0.4920137	0.06743	0.1191000
Original (New Seed)	$\left( 1+d^{\kappa }\right) ^{-1}$	100	0.0000026	0.0000	0.0000000
Original (New Seed)	$\left( 1+d^{\kappa }\right) ^{-1}$	70	0.0000240	0.002046	0.1174000
Original (New Seed)	$\left( 1+d^{\kappa }\right) ^{-1}$	40	0.0009413	0.02326	0.6451000
Original (New Seed)	$\exp \left\{ -\kappa d^{2}\right\}$	100	0.5100904	0.0007158	0.0005900
Original (New Seed)	$\exp \left\{ -\kappa d^{2}\right\}$	70	0.4910087	0.01579	0.05212
Original (New Seed)	$\exp \left\{ -\kappa d^{2}\right\}$	40	0.4991936	0.03086	0.1722

**Fig. 2:**
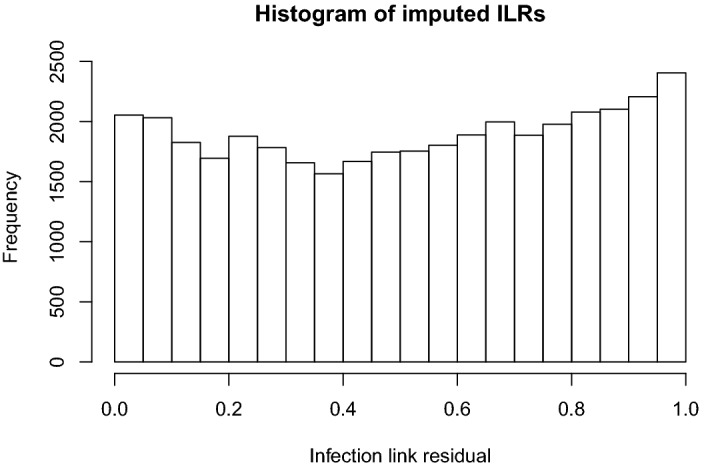
Histogram of imputed ILRs, corresponding to $p\mathrm {-values}<0.05$ for the run where a model with a Gaussian kernel was fitted to data generated with a Exponential kernel and the epidemic was observed until all hosts reached the *I* state

- The ILR based test consistently finds very strong evidence against the model when the power-law kernel is wrongly fitted. However, no evidence emerges when the exponentially bounded, Gaussian kernel is fitted to the observations, with the mean p-value being close to 0.5. This suggests that the ILR test may be insensitive to mis-specification if the degree of discrepancy is modest. When the data are simulated using the larger value of $\kappa$ (so that secondary infection tends to occur over short range) and the power-law kernel is fitted, the evidence against the assumptions is strongest when only 40% of the hosts are infected. This may be due to the short-range secondary infection being most apparent during the early stages of the epidemic where the pattern of infection is clearly formed from isolated foci (caused by primary infection) surrounded by clustered secondary infections. As a result, residuals from the early stage of the epidemic (when the potential choice of exposure locations is widest) may display the greatest evidence against the assumed model and inclusion of residuals from later in the epidemic may serve to dilute this evidence. Simulated epidemics are presented in Electronic Supplementary Material Appendix 3 to illustrate this point.
- In contrast, the LLRT based on the augmented data likelihood elicits some evidence against the assumptions in all cases, including when the Gaussian kernel is fitted. Moreover, in all cases the strength of the evidence as measured by the expected p-value increases as the observation duration increases (and thus the percentage of observed infections).
- The performance of the LLRT that uses the partial likelihood seems variable. It detects evidence in cases where the observation duration is long, but appears to degrade as less of the epidemic is observed. Therefore, the augmented data LLR test may be a more robust approach.
- When we focus on the case where only 40% of the population is infected, we see that the ILR test typically provides most evidence when the power-law kernel is assumed. The augmented data likelihood approach performs best when the Gaussian kernel is assumed. This last observation may illustrate the phenomenon of reinforcement discussed earlier; the imputed data reinforce the ‘wrong’ assumptions of the fitted model and undermine to some extent the capacity of the imputed likelihood ratio test to find evidence against the assumed model when it is poorly specified.
- Although the ILR method fails to detect evidence against the model with a Gaussian kernel, it is nevertheless worth scrutinising the imputed residuals in instances where the *p*-value from the Anderson-Darling test is small, in order to see whether any indication of the mis-specification can be discerned Lau et al. (2014). Figure 2 shows a histogram formed from the union of the imputed sets of residuals for $p < 0.05$. The form of the histogram suggests that these *p*-values are small due to imputed sets of residuals having too many extreme values. Arguably this is consistent with true model being the exponential kernel which qualitatively displays higher infection at short and long ranges then the Gaussian. Interpreting this in the dual observer framework, we might say that, while *B*’s posterior probability that *C* rejects the uniform model for the residuals is low (0.06005004), were *C* to reject it would be likely that this was due to observing too many extreme residuals.

In order to determine whether the tests produce false positives when the fitted kernel is the same as the actual kernel, an extra set of computer runs was performed in which an exponential kernel model was fitted to the simulated data generated from a model with an exponential kernel. The results can be found in Table 3.

The values of $\hat{E(p)}$ in Table 3 show the results of performing a test with $M_{0}$ being the model with the true kernel and $M_{A}$ being an alternative model with a similar spatial kernel. All the tests in the table do not detect discrepancy between the fitted model and the alternative model, thus showing the tests do not have a propensity to produce false positives.

## Conclusions and discussion

In this paper we have investigated methods for assessing and comparing spatio-temporal stochastic epidemic models—particularly with regard to the specification of the spatial kernel function. We have focussed on techniques that avoid the need to increase the complexity of the fitted model (e.g. by specifying more highly parametrised kernel functions). Rather, the methods that we consider can be implemented as relatively straightforward addenda to a Bayesian analysis where the model criticism is achieved by embedding classical testing methods within the Bayesian analysis—in the same spirit as posterior predictive checking. In particular, we have compared the ability of the infection-link residuals introduced by Lau et al. (2014) to detect kernel mis-specification with that of tests based on latent likelihood ratio tests. The simulation study uses data in which the transition into the *I* and *R* states are observed, but can be easily adapted to snapshot data, data with under-reporting and other forms of data censoring (Gamado et al. 2013), where epidemic model selection is often hindered by computational complexity. The results demonstrate that the former approach performs well when the degree of model mis-specification in high - that is when a power-law kernel is assumed when the true kernel is exponential—but is unable to detect evidence when the true and assumed kernels are qualitatively more similar. On the other hand, a test based on an augmented data latent likelihood is able to elicit evidence of the more subtle mis-specification.

**Table 3:** Comparison of Latent Likelihood Ratio (LLR) test to Infection Link Residuals test: data-set, alternative model tested and estimated expected *p*-values from the infection link residuals test, LLR (augmented data likelihood) and LLR (partial likelihood)

Data-set	$M_{A}$	Total % Population Infectious	ILR $\hat{E(p)}$	LLR (augmented data) $\hat{E(p)}$	LLR (Partial) $\hat{E(p)}$
Original	$\exp \left\{ -\kappa d\right\}$	100	0.502260	0.5410	0.649163
Original	$\exp \left\{ -\kappa d\right\}$	70	0.5032322	0.5910	0.6824
Original	$\exp \left\{ -\kappa d\right\}$	40	0.4898694	0.8043	0.7718

The results provide further examples of the phenomenon of reinforcement, whereby applying tests to date are imputed using the model being tested may have little power to detect mis-specification. Since the additional data are imputed using the misspecified model, it need not follow that basing the testing on more data leads to more power. In certain cases, the ILR methodology applied to the emergent phase of the epidemic only provides more evidence of discrepancy than when the data from the full time period is used. This in turns leads to the notion of how best to design a latent experiment, or equivalently select a discrepancy variable based upon imputed data. How can one use prior belief on model parameters to predict (before data are considered) which form of latent test will be best able to detect a suspected mode of mis-specification? Answering this question is a challenge which we seek to address in ongoing work. Nevertheless, we suggest that the techniques presented in this paper can offer readily implementable ways of checking model assumptions while avoiding the complexities and instabilities associated with a purely Bayesian approach.

In this paper, we have opted to focus on methods that augment actual observations with imputed data, so that imputed likelihoods for the augmented data are tractable. An alternative approach would be to seek approximate likelihood functions for the actual data, for example, using the synthetic likelihood approach of Wood (2010), or simulation-based approaches such as ABC (for example, McKinley et al. 2009; Csilléry et al. 2010; Sisson et al. 2018). The synthetic likelihood approach has been used for non-spatial epidemics (Cook et al. 2008), but there are difficulties involved in the use of these methods for the class of models which are the focus of this paper. Exploring these approaches in the context of epidemic models would be of interest. Moment closure methods have already proved useful in providing approximations to likelihoods for non-spatial epidemic models (Krishnarajah et al. 2005, 2007), though extending these approaches to the spatiotemporal setting considered here may be challenging.

### Electronic supplementary material

Below is the link to the electronic supplementary material.

Supplementary material 1 (pdf 638 KB)

## Appendix: impact of imputation on the power of the latent likelihood ratio test

The tests in Sect. 3 may appear to have the potential to be targeted at specific forms of mis-specification but some caveats should be noted. When *B* imputes the latent process *x* in order to specify a tractable classical test, they appeal to the modelling assumptions underlying $M_0$. It follows that imputation will *reinforce* these assumptions to an extent dependent on the amount of imputed information. For example, if the imputed *x* included not only unobserved quantities $x_1$ from the present experiment but also a further $m-1$ independent replicates $x_2, ..., x_m$ then, for large *m*, $\pi (p(\theta , x)| y) \approx \mathrm{U}(0, 1)$ for a large range of tests, since the test result would be increasingly dominated by the imputed replicates (Streftaris and Gibson 2012; Gelman et al. 2013).

To understand the effect of imputation on power more formally, we consider the simple situation where $B's$ prior distribution $\pi _0(\theta )$ places all belief on a single value $\theta _0$, giving a density $\pi _0(x)$ for the latent process *x*. We assume that the alternative model, $M_1$, is simple (i.e. has no free parameters) with sampling distribution $\pi _1(x)$. Suppose now that *B* observes $y = f(x)$, so that *x* is an augmented version of *y* and imputes *x* via $\pi _0( x | y)$. They then impute the p-value, $p_x$ computed by *C* from a likelihood ratio test (LRT) applied to *x*. Suppose that *B* summarises their posterior belief regarding $C's$ evidence against $\pi _0$ by the quantity

$$\begin{aligned} \gamma _{x,\delta }(y) = \pi _0(p_x < \delta | y) \end{aligned}$$

for some suitably small $\delta$. A natural analogue of *power* for *B* would be the expectation of $\gamma _{x,\delta }(y)$ under the alternative hypothesis, that is

$$\begin{aligned} \beta _x = \mathrm{E} \left[ \gamma _{x,\delta }(y) | M_1 \right] . \end{aligned}$$

Note that when $x \equiv y$ the quantity $\gamma _{y,\delta }(y)$ is an indicator function and $\beta _y$ is the power of the uniformly most powerful test obtained using the Neyman-Pearson Lemma. Then we have following result.

### Proposition A.1

For *x*, *y*, $$ M_0$$, $$M_1$$ as described above, $$\beta _x \le \beta _y$$.

### Proof

The most powerful classical test of level $\delta$ of $M_0$ v $M_1$ that can be applied to the imputed *x* is based on the ratio $\frac{\pi ^{i}_0(x)}{\pi ^{i}_1(x)}$ where $\pi ^{i}_0$ and $\pi ^{i}_1$ represent the sampling densities of the *imputed* *x* respectively under $M_0$ and $M_1$. Now $\pi ^{i}_0(x) = \pi _0(y)\pi _0(x|y) = \pi _0(x)$ while $\pi _1(x) = \pi _1(y)\pi _0(x|y)$, so that

$$\begin{aligned} \frac{\pi ^{i}_0(x)}{\pi ^{i}_1(x)} = \frac{\pi _0(y)}{\pi _1(y)}. \end{aligned}$$

Therefore, a LRT applied directly to *y* is equivalent to a LRT applied to *x* when $x \sim \pi ^{i}_0(x)$ and $x \sim \pi ^{i}_1(x)$ are used as the sampling densities of *x* under the competing hypotheses. We denote by $p_y$ the resulting p-value.

Now, for the latent likelihood ratio test, *B* imputes the result of $C's$ likelihood ratio test applied to the imputed *x*, where $C's$ test is based on the test statistic

3 $$\begin{aligned} \frac{\pi _0(x)}{\pi _1(x)} = \frac{\pi ^{i}_0(x)}{\pi _1(x)} \end{aligned}$$

with the associated p-value, $p_x$. By the Neyman-Pearson Lemma, the power of this test cannot exceed that of the optimal test. We therefore have that for any given value $\delta$,

$$\begin{aligned} \Pr (p_x< \delta | M_1) \le \Pr (p_y < \delta | M_1) = \beta _y \end{aligned}$$

where $x \sim \pi ^{i}_1(x)$ and $y \sim \pi _1(y)$ on the left and right-hand sides respectively. Note that

$$\begin{aligned} Pr(p_x< \delta | M_1) = \int \pi _0(p_x < \delta | y) \pi _1(y)dy = \beta _x. \end{aligned}$$

This completes the proof. $\square$

Now suppose more generally that $M_0$ uses an arbitrary prior $\pi _0(\theta )$, while $M_1$ remains simple, and define $\beta _x(\theta )$ and $\beta _y(\theta )$ in the obvious way. Then under the prior distribution, $\beta _y(\theta )$ is absolutely dominant over $\beta _x(\theta )$ so that *B* views with certainty the LRT applied directly to *y* as giving the more powerful test of $M_0$ against $M_1$.

In the above proof the inequality $\beta _x \le \beta _y$ arises from the disparity between $\pi ^{i}_1(x)$ and $\pi _1(x)$. We show that this disparity, as characterised using Kullback-Leibler (KL) divergence, increases as the amount of imputation grows. Suppose that $y = f(x)$ and $x = g(z)$, so that *z* represents the outcome of an experiment that is even more informative than *x*. Consider again the case of simple hypotheses for $M_0$ and $M_1$, with $\pi _0$ and $\pi _1$ denoting the respective sampling densities of quantities.

Repeating the argument used in the proof of the proposition, we note that the optimal LRT based on the imputed *z* would have to use the sampling density of the imputed *z* under the alternative hypothesis, $\pi ^i_{1}(z) = \pi _1(y)\pi _0(z|y)$ resulting in the analytically intractable test statistic $\frac{\pi _0(y)}{\pi _1(y)}$. Our (sub-optimal) LLRT uses the density $\pi _1(z)$ in place of $\pi ^i_{1}(z)$. Note that $\pi _0^i = \pi _0$. We now consider the Kullback-Leibler divergence between $\pi _1^i(z)$ and $\pi _1(z)$. This can be calculated as

$$\begin{aligned} KL(\pi _1^i, \pi _1)= & {} \int \pi _1^i(z) \log (\frac{\pi _1^i(z)}{\pi _1(z)}) dz\\= & {} \int \pi _1^i(z) \log (\frac{\pi _1(y)\pi _0(x|y)\pi _0(z|x)}{\pi _1(y)\pi _1(x|y)\pi _1(z|x)}) dz\\= & {} \int \pi _1^i(z) (\log (\frac{\pi _0(x|y)}{\pi _1(x|y)}) + \log (\frac{\pi _0(z|x)}{\pi _1(z|x)}))dz\\= & {} \int \pi _1^i(z) \log (\frac{\pi _0(x|y)}{\pi _1(x|y)})dz + \int \pi _1^i(z) \log (\frac{\pi _0(z|x)}{\pi _1(z|x)})dz\\= & {} \int \pi _1(y)\pi _0(x|y)\pi _0(z|x)\log (\frac{\pi _1(y)\pi _0(x|y)\pi _0(z|x)}{\pi _1(y)\pi _1(x|y)\pi _0(z|x)})dz\\&+ \int \pi _1(y)\pi _0(x|y)\pi _0(z|x) \log (\frac{\pi _1(y)\pi _0(x|y)\pi _0(z|x)}{\pi _1(y)\pi _0(x|y)\pi _1(z|x)})dz \end{aligned}$$

The first integral above is the KL divergence between the density $\pi _1^i(z) = \pi _1(y)\pi _0(x|y)\pi _0(z|x)$ and the density $\pi _1(y)\pi _1(x|y)\pi _0(z|x)$. Suppose that the latter is used on the denominator in a ratio test statistic applied to the imputed *z*. Then this ratio is clearly $\frac{\pi _0(x)}{\pi _1(x)}$ where *x* is the imputed value and the power of the test corresponds to that of a latent likelihood ratio test applied to *x*. The second integral above is itself a KL divergence greater than zero. It follows that

$$\begin{aligned} KL(\pi _1^i(z), \pi _1(z)) > KL(\pi _1^i(z), \pi _1(y)\pi _1(x|y)\pi _0(z|x)). \end{aligned}$$

In the light of this increasing divergence, we may suspect that the power of a LRT that uses $\pi _1(z)$ on the denominator may be less than that of a test using $\pi _1(y)\pi _1(x|y)\pi _0(z|x)$ or, equivalently, a latent likelihood ratio test applied directly to the imputed *x*. When seeking a suitable latent process *x*, it may be prudent to minimise the extent of imputation and, consequently, the degree of reinforcement of the model under test. That is, if *y* is specified by *x* which, in turn, is specified by *z*, then, assuming the likelihoods $\pi _0(x| \theta )$ and $\pi _1(x | \theta )$ are tractable, *x* should be preferred to *z* as the choice for the latent process.
