# Supplementary material for: Latent likelihood ratio tests for assessing spatial kernels in epidemic models
Source: J Math Biol. 2020 Sep 5;81(3):853–73. doi: 10.1007/s00285-020-01529-3 (PMC7519007; doi:10.1007/s00285-020-01529-3)
Supplement: Supplementary file 1 — Supplementary material 1 (pdf 638 KB) [file 285_2020_1529_MOESM1_ESM.pdf]

Electronic Supplementary Material

David Thong, George Streftaris, Gavin J Gibson

Friday 6th September, 2019

1 Details of MCMC algorithm

1.1 Likelihood

The likelihood can be expressed in the form:

$$
\begin{aligned}
L(\alpha, \beta, \kappa, \mu_E, \sigma_E^2, \mu_I, \sigma_I^2 | \mathbf{x}) \quad \propto \quad & \left(\prod_{i; t_E^{(i)} \leq T} \exp \left[- \int_0^{t_E^{(i)}} C(x_i, t)\, dt \right] \cdot C(x_i, t_E^{(i)}) \right) \cdot \\
& \left(\prod_{i; t_E^{(i)} > T} \exp \left[- \int_0^{T} C(x_i, t)\, dt \right] \right) \cdot \\
& \left(\prod_{i; t_I^{(i)} \leq T} f_E(t_I^{(i)} - t_E^{(i)}; \mu_E, \sigma_E) \right) \cdot \\
& \left(\prod_{i; t_I^{(i)} > T > t_E^{(i)}} \left(1 - F_E(T - t_E^{(i)}; \mu_E, \sigma_E) \right) \right) \cdot \\
& \left(\prod_{i; t_R^{(i)} \leq T} f_I(t_R^{(i)} - t_I^{(i)}; \mu_I, \sigma_I^2) \right) \cdot \\
& \left(\prod_{i; t_R^{(i)} > T > t_I^{(i)}} \left(1 - F_I(T - t_I^{(i)}; \mu_I, \sigma_I^2) \right) \right).
\end{aligned}
$$

1.2 Calculation of the Posterior Expected Imputed P-Value

The process for the computation of the expected posterior imputed p-value is embedded within the data augmented MCMC (a special case of RJMCMC, and is sometimes referred to herein as RJMCMC).

1.2.1 Data Augmented MCMC

The algorithm used for data augmented MCMC (sometimes referred as RJMCMC herein; DAMCMC is a special case of RJMCMC) is:

For each iteration, the following process is repeated:

1. Let the current state of the algorithm at time $k = 0, 1, 2, \ldots$ be denoted by $(\boldsymbol{\theta}^{(k)}, \mathrm{z}^{(k)})$ where $\boldsymbol{\theta}^{(k)} = (\alpha^{(k)}, \beta^{(k)}, \kappa^{(k)}, \mu_E^{(k)}, \sigma_E^{2(k)}, \mu_I^{(k)}, \sigma_I^{2(k)})$ and the times of transition into the exposed state be denoted by $\mathrm{z}^{(k)} = z_1^{(k)}, z_2^{(k)}, \ldots, z_N^{(k)}$ where N is the number of hosts where N is fixed (and hence the length of $z^{(k)}$ is fixed).

2. Using the Metropolis-Hastings algorithm, update each of the parameters in the parameter vector individually. That is, for each parameter $\phi^{(k)}$ in $\boldsymbol{\theta}^{(k)}$:

   (a) Draw a proposal value ϕ' from the proposal distribution $q(\phi'|\phi^{(k)})$.

   (b) Calculate the probability of acceptance α. Let $\boldsymbol{\theta}^*$ be $\boldsymbol{\theta}_t$ with $\phi^{(k)}$ replaced by ϕ':

   $$\alpha = \min\left(1, \frac{\pi(\boldsymbol{\theta}^*|\mathrm{z}^{(k)}, \mathrm{y}) \cdot q(\phi'|\phi^{(k)})}{\pi(\boldsymbol{\theta}^{(k)}|\mathrm{z}^{(k)}, \mathrm{y}) \cdot q(\phi^{(k)}|\phi')}\right)$$

   (c) With probability α, set $\boldsymbol{\theta}^{(k+1)} = \boldsymbol{\theta}^*$, otherwise set $\boldsymbol{\theta}^{(k+1)} = \boldsymbol{\theta}^{(k)}$.

3. For each data item $z_i^{(k)}$ in $\mathrm{z}^{(k)}$:

   (a) **if** $t_I^{(i)} < T$ set move type to *Standard*.

   (b) **else**:

      i. **if** $z_i^{(k)}$ does not fall within $[0, T]$ set move type to *Addition*

      ii. **else** set move type to *Shift* or *Deletion* with probability $\frac{1}{2}$

   (c) **if** move type is *Standard, Addition* or *Shift*: Generate proposal $z_i^{(k)*} \sim \mathrm{Unif}(0, T)$

   (d) **else** set proposed value of $z_i^{(k)*}$to be outside $[0, T]$

   (e) **if** move type is *Standard* or *Shift* set $\nu = 1$

   (f) **else if** move type is *Addition* $\nu = \frac{T}{2}$

   (g) **else if** move type is *Deletion* set $\nu = \frac{2}{T}$

   (h) Set $\mathrm{z}^* = z_1^{(k)}, \ldots, z_i^{(k)*}, \ldots, z_N^{(k)}$. Then the acceptance probability α is given by:

   $$\alpha = \min\left(1, \frac{\pi(\boldsymbol{\theta}^{(k)}|\mathrm{z}^*, \mathrm{y})}{\pi(\boldsymbol{\theta}^{(k)}|\mathrm{z}^{(k)}, \mathrm{y})} \cdot \nu\right)$$

   (i) With probability α, set $\mathrm{z}^{(k+1)} = \mathrm{z}^*$ otherwise $\mathrm{z}^{(k+1)} = \mathrm{z}^{(k)}$

The above algorithm is modified to make it more efficient, by using an independence sampler for the proposal distributions for z_i for the cases where $I_i < T$. Thus, in this case the proposal distribution and acceptance ratio (for the *Standard* moves in Step 3 above) is [7, 3]:

$$q(I_i - z_i, I_i - z_i^*) \equiv \mathrm{Gamma}(\mu_I, \sigma_I^2)$$

$$\alpha = \min\left(1, \frac{\pi(\boldsymbol{\theta}^{(k)}|\mathrm{z}^*, \mathrm{y})}{\pi(\boldsymbol{\theta}^{(k)}|\mathrm{z}^{(k)}, \mathrm{y})} \cdot \frac{q(\mathrm{z}^*|\mathrm{z}^{(k)})}{q(\mathrm{z}^{(k)}|\mathrm{z}^*)}\right)$$

for the cases where it is known that $I_i < T$.

1.2.2 Embedding the Tests within DAMCMC

To embed the tests within the data augmented MCMC, the following steps were added to the algorithm above as an extra step:

4. If k mod $K = 0$ where K is a positive integral value chosen by the user, calculate the test statistic(s).
5. From each test statistic, calculate its p-value and store the p-value obtained.

The following sections describe the calculation of the test statistics and their relevant p-values based upon the full data x which includes both the observed data y and imputed data z.

1.2.3 Infection Link Residuals (ILR) Test Statistic and Imputed P-Value Calculation

Calculation of Test Statistic The infection link residual test is embedded with the RJMCMC and calculated by the following algorithm (from [4]):

1. The infection link for the kth exposure between individuals i and j is chosen with probability p_{ij} from the possible links at time t_k. Primary infection is treated as being an infection caused by a notional infector with force of infection α.
2. The infection links are then ordered and the ranking s' of p_{ij} is determined.
3. Generate a random deviate from Unif($\sum_{l=1}^{s'-1} p_{(l)}, \sum_{l=1}^{s'} p_{(l)}$). This is the imputed infection link residual for the kth exposure.

Calculation of Imputed P-Value The p-value is calculated using the Anderson-Darling test [1]. This is a frequentist test of the hypotheses:

$$H_0 : \text{The data have cumulative distribution function } F(x)$$
$$H_A : \text{The data does not have cumulative distribution function } F(x)$$

The data for this test is a random sample denoted $\{X_1, X_2, \ldots, X_n\}$
Let the empirical distribution function be defined as:

$$F_n(x) = \frac{\text{number of } X_1, X_2 \ldots, X_n \text{ that are} \leq x}{n}$$

The test statistic is defined as:

$$A_n = -n - \frac{1}{n}\sum_{i=1}^{n}(2i-1)\left[\ln F(X_i) + ln(1 - F(X_{n+1-i}))\right] \tag{1}$$

The Anderson-Darling test statistic can be expressed in another form, which shows that it is the integral of the weighted squared difference between the empirical distribution function and the hypothesised distribution function, multiplied by a weighting with weight concentrated towards the tails of the distribution.

$$A_n = n\int_0^1 \frac{[F_n(x) - F(x)]^2}{F(x)(1-F(x))} dF(x) \tag{2}$$

This makes the Anderson-Darling test more able to detect discrepancy between the hypothesised distribution and the data and the tails of the distribution than the Kolmogorov-Smirnov test which is more commonly used.

In this case $F(x)$ is a uniform CDF between 0 and 1, the test statistic simplifies to:

$$A_n = -n - \frac{1}{n}\sum_{i=1}^{n}(2i-1)\left[\ln X_i + ln(1 - X_{n+1-i})\right] \tag{3}$$

1.2.4 Latent Likelihood Ratio Test (LLR) Test Statistic and Imputed P-Value Calculation

Calculation of the Imputed P-Value Without loss of generality, suppose the test statistic used is $T(x,\theta)$ (the same method is used for $T_{partial}(x,\theta)$, except $T(x,\theta)$ is replaced in the following steps with $T_{partial}(x,\theta)$).

1. Calculate $T(x,\theta)$
2. For n_{test} times:
   (a) Generate new data-set $\mathbf{x}^*$ under the fitted model given $\boldsymbol{\theta}^{(k)}$
   (b) Evaluate $T'(\boldsymbol{\theta}^{(k)};\mathbf{x}^*)$
3. Store $\hat{p} = \frac{\text{Count}(T'(\boldsymbol{\theta}^{(k)};x^*)>T(\boldsymbol{\theta}^{(k)};,x))}{n_{test}}$

In practice, it is only practical to have n_{test} set to 1, as this process takes a large amount of time (data-regeneration, and maximum-likelihood estimation takes a relatively large amount of time). Under different values of n_{test}, many different distributions of $\hat{p}$ will be obtained, however, the average of overall MCMC samples of $\hat{p}$ will converge to the expectation of posterior latent p-value regardless of the value of n_{test}. In this paper, this expected value is used to summarise the whole the distribution because of computing power constraints.

2 Maximum Likelihood Estimation Methods

This paper is concerned with the case where the transitions of hosts from the S state to the E state are unobserved, which is often the case in real world epidemics, for example, where there is a latent period (for example, [6, 8]). However, if the transitions to the E state are observed, then since the likelihood function would be tractable in this situation, maximum-likelihood estimation can be used to obtain parameter estimates. In this paper, maximum-likelihood estimation will be embedded within our framework of model testing. This section details how maximum-likelihood estimation is performed within this paper.

2.1 Maximum-Likelihood Estimation of Parameters for Complete Data

In this paper we have used the Subplex algorithm for maximum likelihood estimation, which is heavily based on the Nelder-Mead Algorithm [5]. As in the numerical literature, the optimisation algorithms presented here in this paper are in the form used for function minimisation, but the same algorithm can be used for maximisation since maximisation of a function is the minimisation of the negative of the function. The

Nelder-Mead Simplex algorithm is a gradient-free optimisation algorithm for multivariate functions. The algorithm was chosen because of its flexibility, and inclusion in many programming libraries (for example [2]).

Gradient-based optimisation algorithms can be used in place of the non-gradient based methods used here, although there is a severe drawback in terms of maintaining and debugging the code for separate gradient calculations. Note that the maximisation of the likelihood is performed with bound constraints on the parameter values, which limits the choice of algorithms to those which can handle bound constraints.

Algorithm 1 (Nelder-Mead Simplex Algorithm for function minimisation). *For a real valued function* $f(\mathbf{x}) : x \in \mathbb{R}^n$*, let* $\rho > 0, \chi > \rho, 0 < \gamma < 1, 0 < \sigma < 1$ *(note that in almost all implementations* $\rho = 1, \chi = 2, \gamma = \frac{1}{2}, \sigma = \frac{1}{2}$*). Then for each iteration k, perform the following steps:*

Sort For each vertex in simplex Δ_k*, order the vertices* $\{\mathbf{x}_1, \ldots, \mathbf{x}_{n+1}\}$ *such that*

$$f(\mathbf{x}_1) \leq f(\mathbf{x}_2) \leq \ldots \leq f(\mathbf{x}_{n+1})$$

Reflection Let:

$$\begin{aligned}\bar{\mathbf{x}} &= \frac{\sum_{i=1}^{n} x_i}{n} \\ \mathbf{x}_r &= \bar{\mathbf{x}} + \rho(\bar{\mathbf{x}} - \mathbf{x}_{n+1})\end{aligned}$$

Then, if $f(\mathbf{x}_1) \leq f(\mathbf{x}_r) < f(\mathbf{x}_n)$, $\mathbf{x}_{n+1} = \mathbf{x}_r$

Expansion If $f(\mathbf{x}_r) < f(\mathbf{x}_1)$*, let:*

$$\mathbf{x}_e = \bar{\mathbf{x}} + \chi(\mathbf{x}_r - \bar{\mathbf{x}}) = \bar{\mathbf{x}} + \chi\rho(\bar{\mathbf{x}} - \mathbf{x}_{n+1})$$

If $f(\mathbf{x}_e) < f(\mathbf{x}_r)$ *then* $\mathbf{x}_{n+1} = \mathbf{x}_e$ *else* $\mathbf{x}_{n+1} = \mathbf{x}_r$

Outside Contraction If $f(\mathbf{x}_n) \leq f(\mathbf{x}_r) < f(\mathbf{x}_{n+1})$*, let*

$$\mathbf{x}_c = \bar{\mathbf{x}} + \gamma(\mathbf{x}_r - \bar{\mathbf{x}}) = \bar{\mathbf{x}} + \gamma\rho(\bar{\mathbf{x}} - \mathbf{x}_{n+1})$$

If $f(\mathbf{x}_c) < f(\mathbf{x}_r)$ *then* $\mathbf{x}_{n+1} = \mathbf{x}_c$ *and go to next iteration* $k + 1$*, else* $\mathbf{x}_{n+1} = \mathbf{x}_r$ *and go to shrink step*

Inside Contraction If $f(\mathbf{x}_r) \geq f(\mathbf{x}_{n+1})$*, let*

$$\mathbf{x}_{cc} = \bar{\mathbf{x}} - \gamma(\bar{\mathbf{x}} - \mathbf{x}_{n+1})$$

If $f(\mathbf{x}_{cc}) < f(\mathbf{x}_r)$ *then* $\mathbf{x}_{n+1} = \mathbf{x}_{cc}$ *and go to next iteration* $k + 1$*, else* $\mathbf{x}_{n+1} = \mathbf{x}_r$ *and go to shrink step*

Shrink Let

$$\mathbf{x}_i = \mathbf{x}_1 + \sigma(\mathbf{x}_i - \mathbf{x}_1)$$

for all $i > 1$.

2.1.1 The Subplex Algorithm for Maximisation

The Subplex algorithm incorporates the Nelder-Mead algorithm within itself, and intends to improve convergence to the actual maximum. A brief formal outline is as follows (as described in [9]):

Algorithm 2 (Subplex). *In addition to the Nelder-Mead coefficients defined above (the default Nelder-Mead strategy used for Nelder-Mead algorithm embedded in the Subplex algorithm is* $\rho = 1, \chi = 2, \gamma = \frac{1}{2}, \sigma = \frac{1}{2}$*), let "scale" be a vector of step sizes. Let* ψ, ω *be the simplex reduction coefficients and step reduction coefficients respectively where* $0 < \psi < 1$ *and* $0 < \omega < 1$*. Let nsmin and nsmax be the minimum and maximum subspace dimensions. The default values of these settings (used in this paper) are* $\psi = 0.25, \omega = 0.1, nsmin = \min(2, n), nsmax = \min(5, n)$*. Let* $\mathbf{x}$ *be the current approximation to the minimum.*

1. Determine step size (see Algorithm 3).
2. Set subspaces (see Algorithm 4).
3. Perform the Nelder-Mead algorithm on each subspace.
4. If

$$\frac{\max\left(\|\Delta\mathbf{x}\|_\infty, \|step \cdot \psi\|_\infty\right)}{\max(\|x\|_\infty, 1)} < tol$$

   where Δx is the difference between x and its value on the previous cycle of the algorithm, and *tol* is the error tolerance required, then the algorithm ends. Otherwise, go to step 1.

Algorithm 3 (Subplex (Setting the Step-size)).

1. If the algorithm has just been started, that is, this is the first time that the step-size is being set, $step = scale$. Otherwise, perform the following steps:

   (a) $$step \leftarrow \begin{cases} step \cdot \min\left(\max\left(\frac{\|\Delta\mathbf{x}\|_1}{\|step\|_1}, \omega\right), \frac{1}{\omega}\right) & nsubs > 1 \\ step \cdot \psi & nsubs = 1 \end{cases}$$

   (b) for each component $step_i$ of *step*:

$$step_i \leftarrow \begin{cases} \text{sign}(\Delta x_i) \cdot |step_i| & \Delta x_i \neq 0 \\ -step_i & \Delta x_i = 0 \end{cases}$$

where $\|\mathbf{x}\|_1 = \sum_i |x_i|$.

Algorithm 4 (Subplex (Setting the subspaces)). *Let* $\Delta\mathbf{x}$ *be the vector of progress. Let nsmin and nsmax be the minimum and maximum subspace dimensions. Let there be subs subspaces of* $ns_1, ns_2, \ldots ns_{subs}$ *dimensions (which sum to n), where* $\forall i \in \{1, 2, \ldots, subs\} : nsmin \leq ns_i \leq nsmax$*.*

1. Sort $\Delta\mathbf{x} = (\Delta x_1, \Delta x_2, \ldots, \Delta x_n)$ such that the largest component is first. Let this be denoted $\widetilde{\Delta\mathbf{x}} = (\widetilde{\Delta x}_1, \widetilde{\Delta x}_2, \ldots, \widetilde{\Delta x}_n)$.
2. Set

$$ns_1 = \arg\max_{k \in K} \left(\begin{cases} \frac{\|(\widetilde{\Delta x}_1, \ldots, \widetilde{\Delta x}_k)\|_1}{k} - \frac{\|(\widetilde{\Delta x}_{k+1}, \ldots, \widetilde{\Delta x}_n)\|_1}{n-k} & k \neq n \\ \frac{\|(\widetilde{\Delta x}_1, \ldots, \widetilde{\Delta x}_k)\|_1}{k} & k = n \end{cases} \right)$$

   where $K = \{k | nsmin \leq k \leq nsmax$ and $nsmin \lceil (n-k)/nsmax \rceil \leq n - k\}$, $\|\mathbf{x}\|_1 = \sum_i |x_i|$. This step determines where the "gaps" are in $\widetilde{\Delta\mathbf{x}}$.

3. Repeat step 2 for $ns_2, ns_3, \ldots$ until $\sum_{i=1}^{subs} = n$.

An advantage of the simplex algorithm is that it allows bound constraints on values of $\mathbf{x}$ (see [9], [2]). If any of the Nelder-Mead steps produces a simplex with a point outside of these constraints, the point is moved (usually to the nearest point within the constraints) such that the simplex lies within the constraints.

2.2 Maximum Likelihood Estimation and Model Comparison Methods

We use the following algorithm for each maximum likelihood estimation (performed in bulk) within the algorithms (for model comparison) in this paper:

1. Utilise the optimisation algorithm (Subplex) to obtain an estimate of the MLE (within given stopping criteria and tolerances)
2. Verify that this is the maximum likelihood estimate by restarting the optimiser at a different starting point. If the optimiser produces a point with the same likelihood value (subject to tolerances that have been pre-specified) accept the current estimate as the maximum likelihood estimate. Otherwise, take the point which produces the largest likelihood value and repeat this process again from the start.

3 Examples of Simulated Epidemics

Plots of the data-sets in the simulation study in Section 5 of the paper can be found in Figures 1, 2, 3 and 4.

4 Initialisation Values for MCMC

The algorithm was initialised with $\mathbf{z}^{(0)}$ equal to the true exposure times, and parameter values $\boldsymbol{\theta}^{(0)}$ set to the MLEs of $\boldsymbol{\theta}$ given $\mathbf{z}^{(0)}$ and observed data y. Test runs show that this gives fast convergence to the stationary distribution, whilst converging to the same distribution obtained when setting the starting values of $\mathbf{z}^{(0)}$ to uniformly distributed values between 0 and entry time into the I state, and parameter values $\boldsymbol{\theta}^{(0)}$ set to the MLEs of $\boldsymbol{\theta}$ given $\mathbf{z}^{(0)}$ and observed data y.

5 Examples of Posterior Densities

Examples of posterior densities are given in figures 5, 6, 7 and 8. Table 1 shows the details of the MCMC runs plotted and which figures correspond to which computer runs. The trace and density plots obtained for the other MCMC runs were similar to those shown here.

6 Parameter Posterior Summary Statistics

Parameter posterior summary statistics for some of the posterior distributions obtained in the simulation study in Section 5 of the paper can be found in Tables 2,3,4,5,6,7 and 8.

0

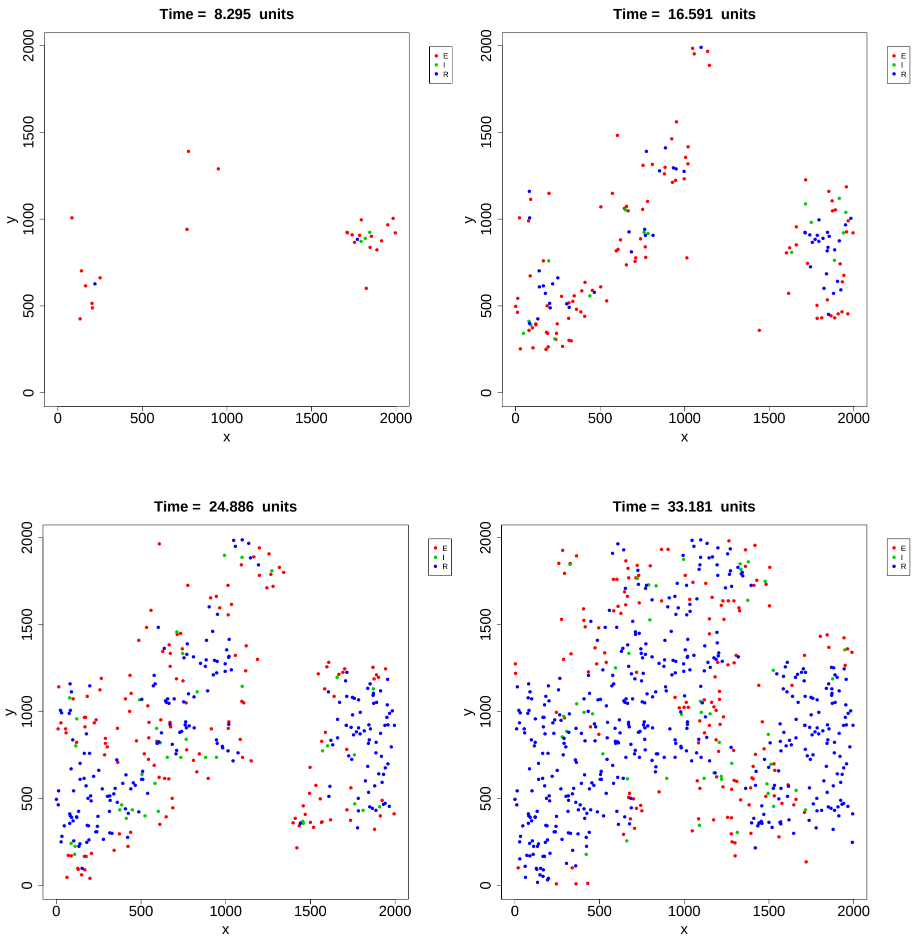

Figure 1: Snapshots of the simulated epidemic dataset "Original", which is generated with an exponential kernel. Each point on the graph represents one host. Points are colour-coded to represent the current state of the host. Susceptible points are not displayed to maintain clarity of the graph. The colour of the points on the graph indicate the state of each host at the given time. Red indicates the host is exposed, green indicates the host is infectious and blue indicates that the host is removed.

Data-set	Kernel Fitted	Total %<br>Infections Observed	Figures
Original	$(1+d^{\kappa})^{-1}$	70	Fig. 7, Fig. 8
Original	$\exp\{-\kappa d^2\}$	70	Fig. 5, Fig. 6

Table 1: Table of runs and the figures displaying their trace and density plots

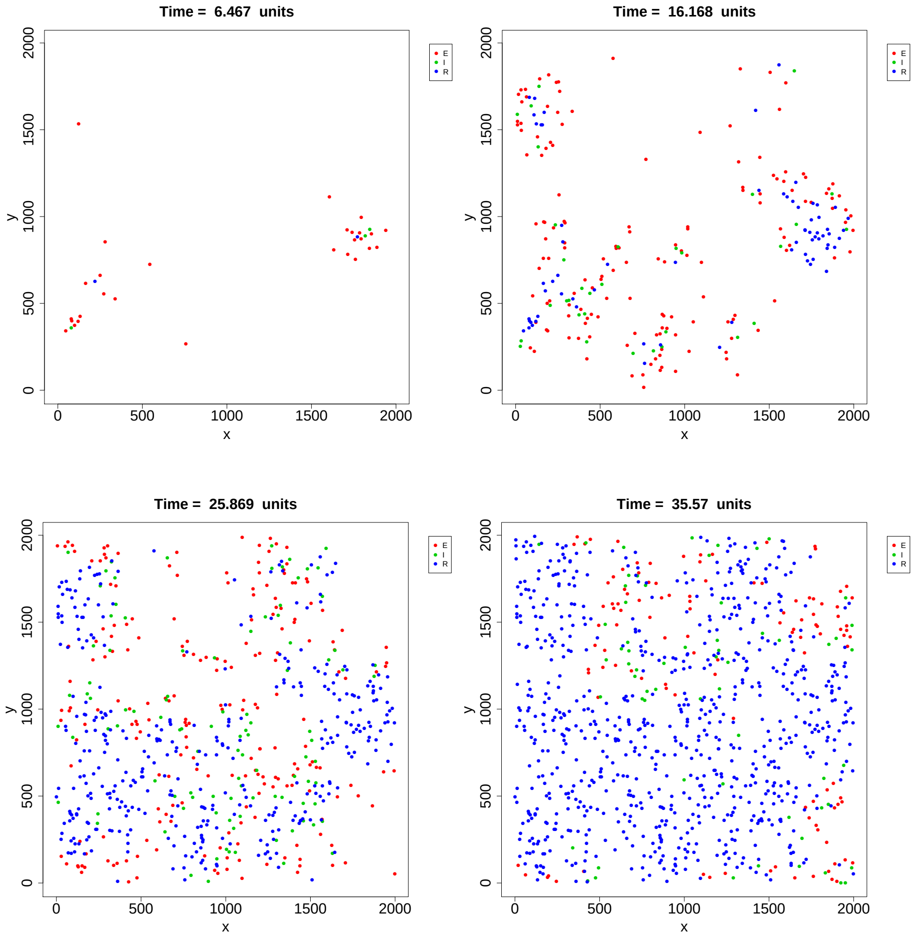

Figure 2: Snapshots of the simulated epidemic dataset $2 \times \alpha$, which is generated with an exponential kernel. Each point on the graph represents one host. Points are colour-coded to represent the current state of the host. Susceptible points are not displayed to maintain clarity of the graph. The colour of the points on the graph indicate the state of each host at the given time. Red indicates the host is exposed, green indicates the host is infectious and blue indicates that the host is removed.

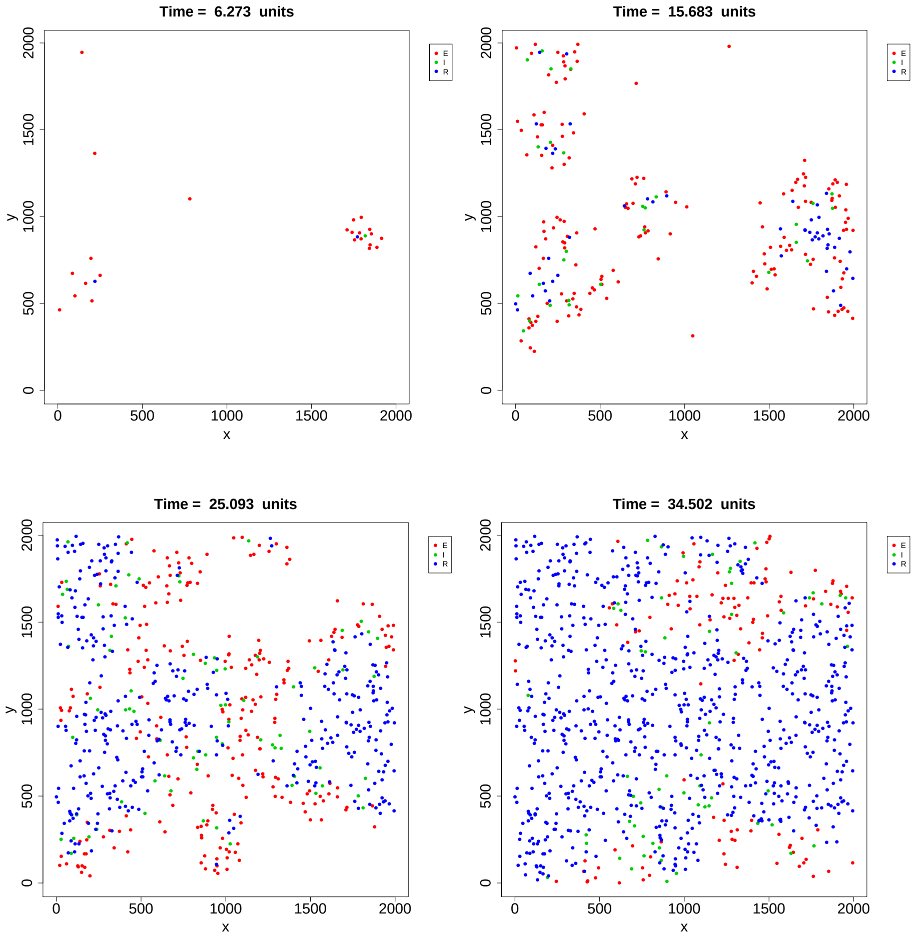

Figure 3: Snapshots of the simulated epidemic dataset $2 \times \beta$, which is generated with an exponential kernel. Each point on the graph represents one host. Points are colour-coded to represent the current state of the host. Susceptible points are not displayed to maintain clarity of the graph. The colour of the points on the graph indicate the state of each host at the given time. Red indicates the host is exposed, green indicates the host is infectious and blue indicates that the host is removed.

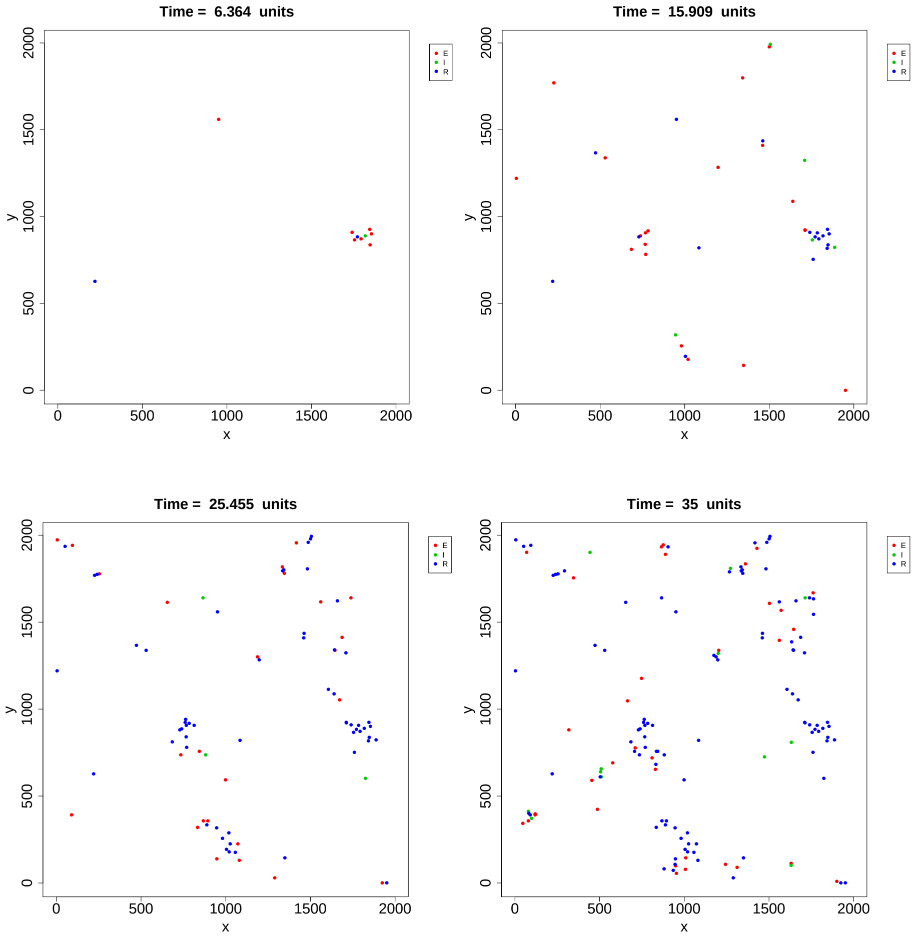

Figure 4: Snapshots of the simulated epidemic dataset $2 \times \gamma$, which is generated with an exponential kernel. Each point on the graph represents one host. Points are colour-coded to represent the current state of the host. Susceptible points are not displayed to maintain clarity of the graph. The colour of the points on the graph indicate the state of each host at the given time. Red indicates the host is exposed, green indicates the host is infectious and blue indicates that the host is removed.

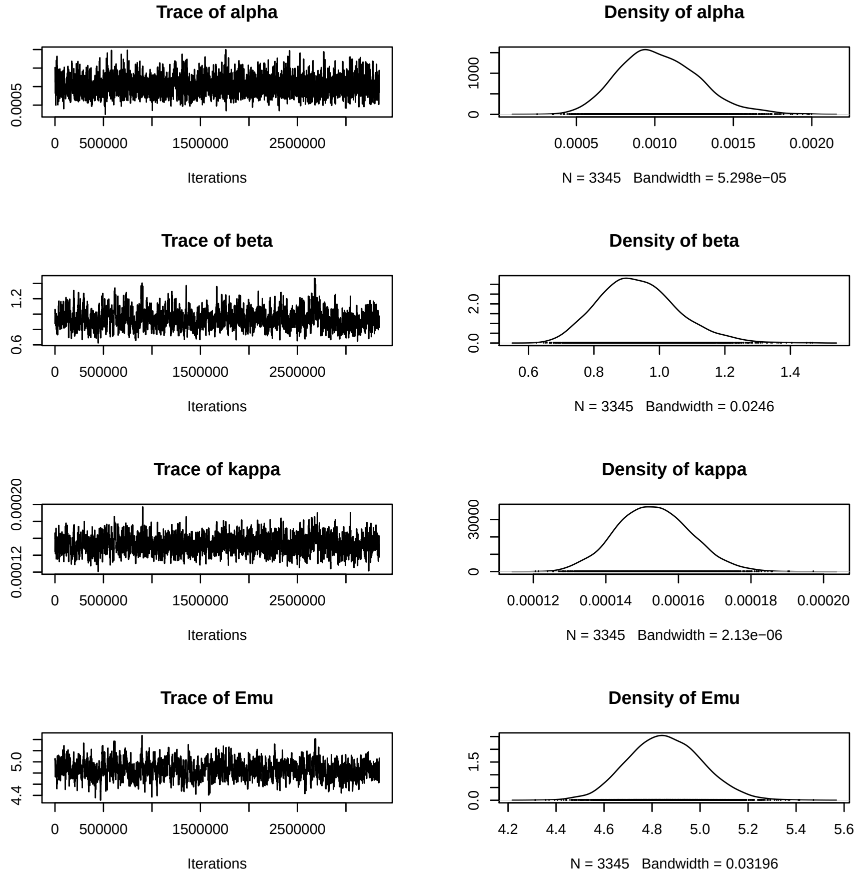

Figure 5: Trace and density plot of MCMC run (see Table 1 for more information)

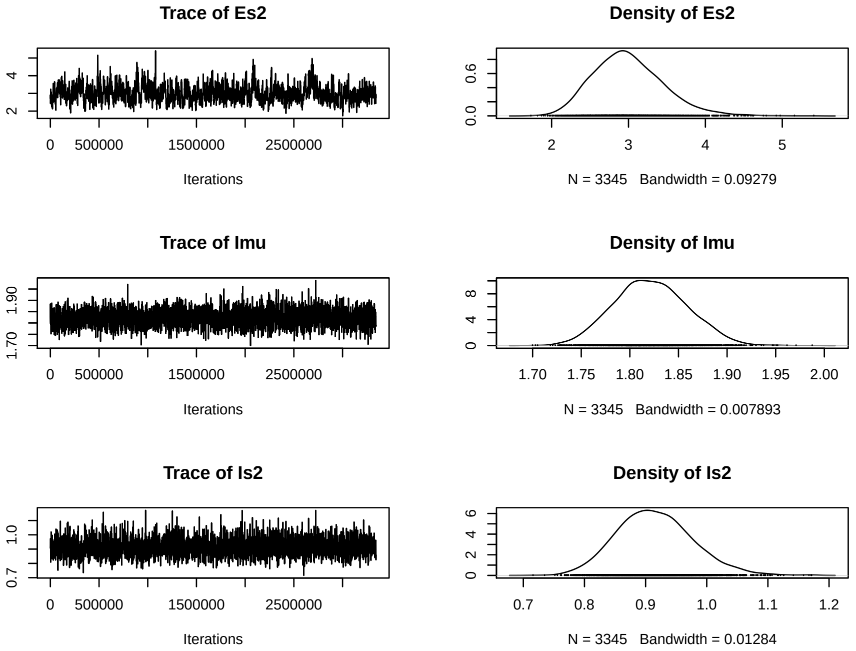

Figure 6: Trace and density plot of MCMC run (see Table 1 for more information)

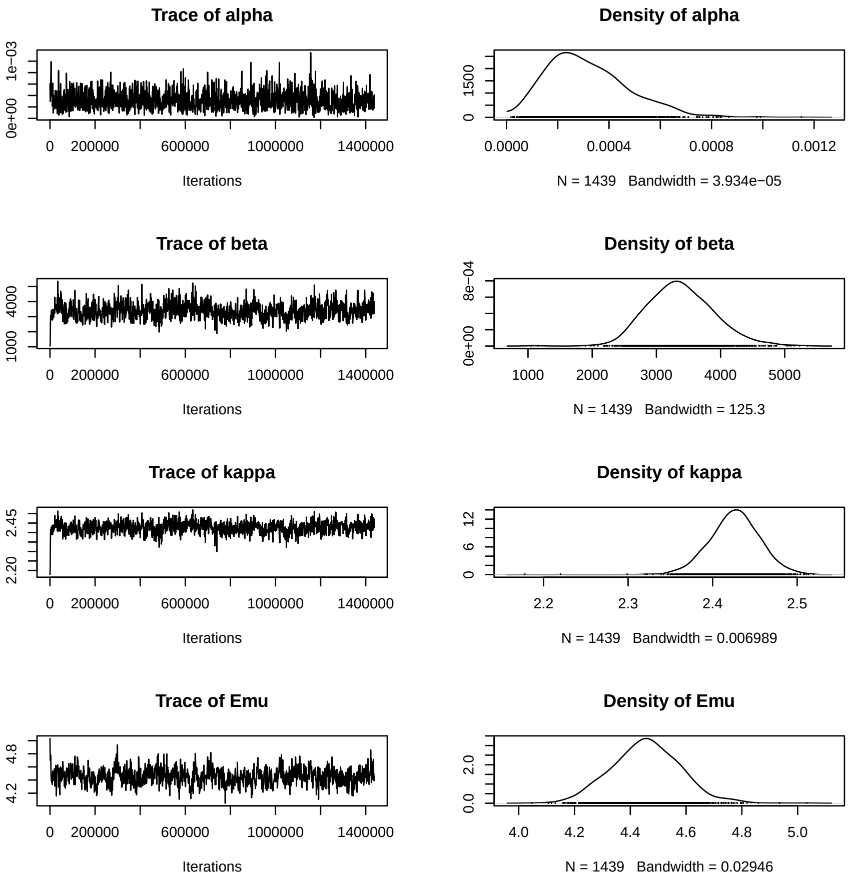

Figure 7: Trace and density plot of MCMC run (see Table 1 for more information)

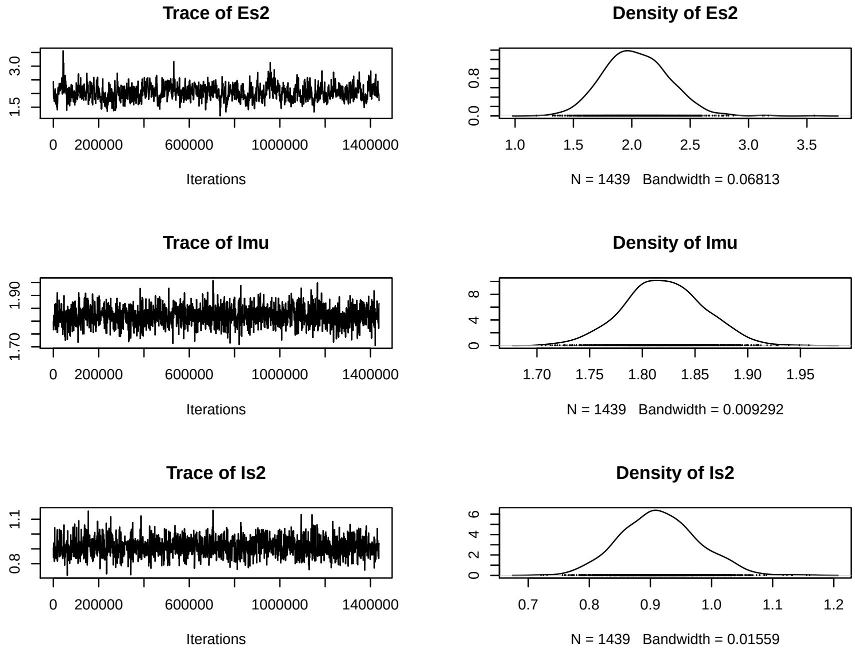

Figure 8: Trace and density plot of MCMC run (see Table 1 for more information)

Data-set	Total % Population Infectious	H_0	α	
			Mean	S.D
$\alpha \times 2$	100	$(1+d^{\kappa})^{-1}$	0.00133	0.0003334
$\alpha \times 2$	70	$(1+d^{\kappa})^{-1}$	0.0006492	0.0002724
$\alpha \times 2$	40	$(1+d^{\kappa})^{-1}$	0.0006613	0.0002828
$\alpha \times 2$	100	$\exp\left\{-\kappa d^2\right\}$	0.0029139	0.0004486
$\alpha \times 2$	70	$\exp\left\{-\kappa d^2\right\}$	0.002556	0.0004495
$\alpha \times 2$	40	$\exp\left\{-\kappa d^2\right\}$	0.0022339	0.0007029
$\beta \times 2$	100	$(1+d^{\kappa})^{-1}$	0.0004442	0.0001923
$\beta \times 2$	70	$(1+d^{\kappa})^{-1}$	0.0003817	0.0001863
$\beta \times 2$	40	$(1+d^{\kappa})^{-1}$	0.0004015	0.0001984
$\beta \times 2$	100	$\exp\left\{-\kappa d^2\right\}$	0.0012901	0.0003207
$\beta \times 2$	70	$\exp\left\{-\kappa d^2\right\}$	0.0011284	0.0003027
$\beta \times 2$	40	$\exp\left\{-\kappa d^2\right\}$	0.001087	0.0003053
$\kappa \times 2$	100	$(1+d^{\kappa})^{-1}$	0.0008384	0.00004733
$\kappa \times 2$	70	$(1+d^{\kappa})^{-1}$	0.0007108	0.00006799
$\kappa \times 2$	40	$(1+d^{\kappa})^{-1}$	0.0005803	0.0001156
$\kappa \times 2$	100	$\exp\left\{-\kappa d^2\right\}$	0.0010206	0.0000484
$\kappa \times 2$	70	$\exp\left\{-\kappa d^2\right\}$	0.0010663	0.00007045
$\kappa \times 2$	40	$\exp\left\{-\kappa d^2\right\}$	0.0012108	0.0001243
Original	100	$(1+d^{\kappa})^{-1}$	0.0003585	0.000178
Original	70	$(1+d^{\kappa})^{-1}$	0.0003146	0.0001575
Original	40	$(1+d^{\kappa})^{-1}$	0.000696	0.000193
Original	100	$\exp\left\{-\kappa d^2\right\}$	0.0013476	0.0002592
Original	70	$\exp\left\{-\kappa d^2\right\}$	0.0010269	0.0002521
Original	40	$\exp\left\{-\kappa d^2\right\}$	0.0009899	0.000265
Original (New Seed)	100	$(1+d^{\kappa})^{-1}$	0.001213	0.0002561
Original (New Seed)	70	$(1+d^{\kappa})^{-1}$	0.0009564	0.0003077
Original (New Seed)	40	$(1+d^{\kappa})^{-1}$	0.001012	0.0003282
Original (New Seed)	100	$\exp\left\{-\kappa d^2\right\}$	0.0016983	0.0002973
Original (New Seed)	70	$\exp\left\{-\kappa d^2\right\}$	0.0016752	0.0003794
Original (New Seed)	40	$\exp\left\{-\kappa d^2\right\}$	0.0018358	0.0004172

Table 2: Table of Posterior Means and Standard Deviation for the simulation study runs for α

Data-set	Total % Population Infectious	H_0	β Mean	β S.D
$\alpha \times 2$	100	$(1+d^{\kappa})^{-1}$	3512	506.4
$\alpha \times 2$	70	$(1+d^{\kappa})^{-1}$	2776	443.8
$\alpha \times 2$	40	$(1+d^{\kappa})^{-1}$	1796	374.7
$\alpha \times 2$	100	$\exp\{-\kappa d^2\}$	0.9023087	0.09043
$\alpha \times 2$	70	$\exp\{-\kappa d^2\}$	0.9037788	0.109
$\alpha \times 2$	40	$\exp\{-\kappa d^2\}$	0.8149542	0.5498334
$\beta \times 2$	100	$(1+d^{\kappa})^{-1}$	4794	635.7
$\beta \times 2$	70	$(1+d^{\kappa})^{-1}$	3678	559.2
$\beta \times 2$	40	$(1+d^{\kappa})^{-1}$	2540	457.7
$\beta \times 2$	100	$\exp\{-\kappa d^2\}$	1.0636219	0.141
$\beta \times 2$	70	$\exp\{-\kappa d^2\}$	0.9717919	0.149
$\beta \times 2$	40	$\exp\{-\kappa d^2\}$	0.8408861	0.1646
$\kappa \times 2$	100	$(1+d^{\kappa})^{-1}$	2894	426.3
$\kappa \times 2$	70	$(1+d^{\kappa})^{-1}$	2538	401.9
$\kappa \times 2$	40	$(1+d^{\kappa})^{-1}$	2149	384.4
$\kappa \times 2$	100	$\exp\{-\kappa d^2\}$	0.9636156	0.07927
$\kappa \times 2$	70	$\exp\{-\kappa d^2\}$	0.9170497	0.08298
$\kappa \times 2$	40	$\exp\{-\kappa d^2\}$	0.9734283	0.1103
Original	100	$(1+d^{\kappa})^{-1}$	2558	476.7
Original	70	$(1+d^{\kappa})^{-1}$	3533	525.7
Original	40	$(1+d^{\kappa})^{-1}$	4313	561.4
Original	100	$\exp\{-\kappa d^2\}$	0.8482083	0.08723
Original	70	$\exp\{-\kappa d^2\}$	0.9342897	0.1178
Original	40	$\exp\{-\kappa d^2\}$	0.9450705	0.1553
Original (New Seed)	100	$(1+d^{\kappa})^{-1}$	3731	540.5
Original (New Seed)	70	$(1+d^{\kappa})^{-1}$	3114	523.6
Original (New Seed)	40	$(1+d^{\kappa})^{-1}$	2129	431.8
Original (New Seed)	100	$\exp\{-\kappa d^2\}$	0.6972467	0.06671
Original (New Seed)	70	$\exp\{-\kappa d^2\}$	0.7186071	0.08508
Original (New Seed)	40	$\exp\{-\kappa d^2\}$	0.8079134	0.122

Table 3: Table of Posterior Means and Standard Deviation for the simulation study runs for β

Data-set	Total % Population Infectious	H_0	κ	
			Mean	S.D
$\alpha \times 2$	100	$(1+d^{\kappa})^{-1}$	2.392	0.02861
$\alpha \times 2$	70	$(1+d^{\kappa})^{-1}$	2.369	0.03153
$\alpha \times 2$	40	$(1+d^{\kappa})^{-1}$	2.284	0.04026
$\alpha \times 2$	100	$\exp\{-\kappa d^2\}$	0.0001535	0.00000898
$\alpha \times 2$	70	$\exp\{-\kappa d^2\}$	0.0001548	0.00001012
$\alpha \times 2$	40	$\exp\{-\kappa d^2\}$	0.0001448	0.0002132
$\beta \times 2$	100	$(1+d^{\kappa})^{-1}$	2.414	0.02534
$\beta \times 2$	70	$(1+d^{\kappa})^{-1}$	2.388	0.02941
$\beta \times 2$	40	$(1+d^{\kappa})^{-1}$	2.312	0.03489
$\beta \times 2$	100	$\exp\{-\kappa d^2\}$	0.0001178	0.000007319
$\beta \times 2$	70	$\exp\{-\kappa d^2\}$	0.0001136	0.000007957
$\beta \times 2$	40	$\exp\{-\kappa d^2\}$	0.0001008	0.000008752
$\kappa \times 2$	100	$(1+d^{\kappa})^{-1}$	2.673	0.0357
$\kappa \times 2$	70	$(1+d^{\kappa})^{-1}$	2.644	0.03832
$\kappa \times 2$	40	$(1+d^{\kappa})^{-1}$	2.604	0.04290
$\kappa \times 2$	100	$\exp\{-\kappa d^2\}$	0.0006301	0.00003171
$\kappa \times 2$	70	$\exp\{-\kappa d^2\}$	0.0006164	0.00003415
$\kappa \times 2$	40	$\exp\{-\kappa d^2\}$	0.0006277	0.00004283
Original	100	$(1+d^{\kappa})^{-1}$	2.378	0.03547
Original	70	$(1+d^{\kappa})^{-1}$	2.434	0.02942
Original	40	$(1+d^{\kappa})^{-1}$	2.455	0.02589
Original	100	$\exp\{-\kappa d^2\}$	0.0001498	0.000008934
Original	70	$\exp\{-\kappa d^2\}$	0.0001527	0.00001006
Original	40	$\exp\{-\kappa d^2\}$	0.0001521	0.00001294
Original (New Seed)	100	$(1+d^{\kappa})^{-1}$	2.41	0.02903
Original (New Seed)	70	$(1+d^{\kappa})^{-1}$	2.377	0.04164
Original (New Seed)	40	$(1+d^{\kappa})^{-1}$	2.296	0.04251
Original (New Seed)	100	$\exp\{-\kappa d^2\}$	0.0001295	0.00000753
Original (New Seed)	70	$\exp\{-\kappa d^2\}$	0.0001275	0.000008302
Original (New Seed)	40	$\exp\{-\kappa d^2\}$	0.0001332	0.00001107

Table 4: Table of Posterior Means and Standard Deviation for the simulation study runs for κ

Data-set	Total % Population Infectious	H_0	μ_E Mean	μ_E S.D
$\alpha \times 2$	100	$(1+d^{\kappa})^{-1}$	4.805	0.1031
$\alpha \times 2$	70	$(1+d^{\kappa})^{-1}$	4.523	0.1189
$\alpha \times 2$	40	$(1+d^{\kappa})^{-1}$	4.451	0.1521
$\alpha \times 2$	100	$\exp\{-\kappa d^2\}$	5.042126	0.1102
$\alpha \times 2$	70	$\exp\{-\kappa d^2\}$	4.9593773	0.1377
$\alpha \times 2$	40	$\exp\{-\kappa d^2\}$	4.843329	0.1968621
$\beta \times 2$	100	$(1+d^{\kappa})^{-1}$	4.289	0.1153
$\beta \times 2$	70	$(1+d^{\kappa})^{-1}$	4.041	0.1357
$\beta \times 2$	40	$(1+d^{\kappa})^{-1}$	4.022	0.1633
$\beta \times 2$	100	$\exp\{-\kappa d^2\}$	4.7652099	0.124
$\beta \times 2$	70	$\exp\{-\kappa d^2\}$	4.6984782	0.1549
$\beta \times 2$	40	$\exp\{-\kappa d^2\}$	4.6826162	0.2359
$\kappa \times 2$	100	$(1+d^{\kappa})^{-1}$	4.945	0.1001
$\kappa \times 2$	70	$(1+d^{\kappa})^{-1}$	4.929	0.1121
$\kappa \times 2$	40	$(1+d^{\kappa})^{-1}$	4.969	0.1349
$\kappa \times 2$	100	$\exp\{-\kappa d^2\}$	4.9826195	0.09697
$\kappa \times 2$	70	$\exp\{-\kappa d^2\}$	4.979419	0.1082
$\kappa \times 2$	40	$\exp\{-\kappa d^2\}$	5.0850028	0.1401
Original	100	$(1+d^{\kappa})^{-1}$	4.42	0.162
Original	70	$(1+d^{\kappa})^{-1}$	4.473	0.1243
Original	40	$(1+d^{\kappa})^{-1}$	4.579	0.1075
Original	100	$\exp\{-\kappa d^2\}$	4.793396	0.1173
Original	70	$\exp\{-\kappa d^2\}$	4.8615001	0.1504
Original	40	$\exp\{-\kappa d^2\}$	4.9118098	0.1972
Original (New Seed)	100	$(1+d^{\kappa})^{-1}$	4.862	0.1082
Original (New Seed)	70	$(1+d^{\kappa})^{-1}$	4.756	0.1305
Original (New Seed)	40	$(1+d^{\kappa})^{-1}$	4.66	0.1573
Original (New Seed)	100	$\exp\{-\kappa d^2\}$	4.9423912	0.1094
Original (New Seed)	70	$\exp\{-\kappa d^2\}$	4.9488673	0.1389
Original (New Seed)	40	$\exp\{-\kappa d^2\}$	4.9545913	0.1762

Table 5: Table of Posterior Means and Standard Deviation for the simulation study runs for μ_E

Data-set	Total % Population Infectious	H_0	σ_E^2	
			Mean	S.D
$\alpha \times 2$	100	$(1+d^{\kappa})^{-1}$	1.956	0.2294
$\alpha \times 2$	70	$(1+d^{\kappa})^{-1}$	1.741	0.2386
$\alpha \times 2$	40	$(1+d^{\kappa})^{-1}$	1.805	0.344
$\alpha \times 2$	100	$\exp\left\{-\kappa d^2\right\}$	2.4812384	0.2898
$\alpha \times 2$	70	$\exp\left\{-\kappa d^2\right\}$	2.3839773	0.3476
$\alpha \times 2$	40	$\exp\left\{-\kappa d^2\right\}$	2.1531098	0.4522831
$\beta \times 2$	100	$(1+d^{\kappa})^{-1}$	1.925	0.2174
$\beta \times 2$	70	$(1+d^{\kappa})^{-1}$	1.819	0.2626
$\beta \times 2$	40	$(1+d^{\kappa})^{-1}$	1.912	0.352
$\beta \times 2$	100	$\exp\left\{-\kappa d^2\right\}$	2.5839779	0.2968
$\beta \times 2$	70	$\exp\left\{-\kappa d^2\right\}$	2.4853798	0.3787
$\beta \times 2$	40	$\exp\left\{-\kappa d^2\right\}$	2.6409302	0.6064
$\kappa \times 2$	100	$(1+d^{\kappa})^{-1}$	2.816	0.2872
$\kappa \times 2$	70	$(1+d^{\kappa})^{-1}$	2.872	0.339
$\kappa \times 2$	40	$(1+d^{\kappa})^{-1}$	2.702	0.3944
$\kappa \times 2$	100	$\exp\left\{-\kappa d^2\right\}$	2.64989	0.2738
$\kappa \times 2$	70	$\exp\left\{-\kappa d^2\right\}$	2.6699676	0.3042
$\kappa \times 2$	40	$\exp\left\{-\kappa d^2\right\}$	2.8226393	0.4221
Original	100	$(1+d^{\kappa})^{-1}$	2.057	0.3825
Original	70	$(1+d^{\kappa})^{-1}$	2.06	0.2758
Original	40	$(1+d^{\kappa})^{-1}$	2.236	0.2442
Original	100	$\exp\left\{-\kappa d^2\right\}$	2.7646615	0.3458
Original	70	$\exp\left\{-\kappa d^2\right\}$	2.9812697	0.4669
Original	40	$\exp\left\{-\kappa d^2\right\}$	2.8702635	0.5652
Original (New Seed)	100	$(1+d^{\kappa})^{-1}$	2.362	0.2636
Original (New Seed)	70	$(1+d^{\kappa})^{-1}$	2.26	0.2975
Original (New Seed)	40	$(1+d^{\kappa})^{-1}$	1.934	0.3628
Original (New Seed)	100	$\exp\left\{-\kappa d^2\right\}$	2.572538	0.3046
Original (New Seed)	70	$\exp\left\{-\kappa d^2\right\}$	2.4965248	0.3723
Original (New Seed)	40	$\exp\left\{-\kappa d^2\right\}$	2.5261648	0.4714

Table 6: Table of Posterior Means and Standard Deviation for the simulation study runs for σ_E^2

Data-set	Total % Population Infectious	H_0	μ_I	
			Mean	S.D
$\alpha \times 2$	100	$(1+d^\kappa)^{-1}$	1.801	0.03027
$\alpha \times 2$	70	$(1+d^\kappa)^{-1}$	1.816	0.03786
$\alpha \times 2$	40	$(1+d^\kappa)^{-1}$	1.825	0.05211
$\alpha \times 2$	100	$\exp\{-\kappa d^2\}$	1.8004701	0.03029
$\alpha \times 2$	70	$\exp\{-\kappa d^2\}$	1.8152959	0.03783
$\alpha \times 2$	40	$\exp\{-\kappa d^2\}$	1.8247609	0.0520868
$\beta \times 2$	100	$(1+d^\kappa)^{-1}$	1.8	0.03024
$\beta \times 2$	70	$(1+d^\kappa)^{-1}$	1.796	0.03717
$\beta \times 2$	40	$(1+d^\kappa)^{-1}$	1.825	0.05158
$\beta \times 2$	100	$\exp\{-\kappa d^2\}$	1.8002562	0.03023
$\beta \times 2$	70	$\exp\{-\kappa d^2\}$	1.7959016	0.03717
$\beta \times 2$	40	$\exp\{-\kappa d^2\}$	1.8250691	0.05174
$\kappa \times 2$	100	$(1+d^\kappa)^{-1}$	1.801	0.0302
$\kappa \times 2$	70	$(1+d^\kappa)^{-1}$	1.835	0.03691
$\kappa \times 2$	40	$(1+d^\kappa)^{-1}$	1.851	0.04849
$\kappa \times 2$	100	$\exp\{-\kappa d^2\}$	1.8003755	0.03025
$\kappa \times 2$	70	$\exp\{-\kappa d^2\}$	1.8351697	0.03694
$\kappa \times 2$	40	$\exp\{-\kappa d^2\}$	1.8512757	0.04859
Original	100	$(1+d^\kappa)^{-1}$	1.798	0.05017
Original	70	$(1+d^\kappa)^{-1}$	1.821	0.03738
Original	40	$(1+d^\kappa)^{-1}$	1.8	0.0303
Original	100	$\exp\{-\kappa d^2\}$	1.8003004	0.03019
Original	70	$\exp\{-\kappa d^2\}$	1.8210957	0.03744
Original	40	$\exp\{-\kappa d^2\}$	1.797982	0.05013
Original (New Seed)	100	$(1+d^\kappa)^{-1}$	1.752	0.02889
Original (New Seed)	70	$(1+d^\kappa)^{-1}$	1.771	0.03564
Original (New Seed)	40	$(1+d^\kappa)^{-1}$	1.793	0.04971
Original (New Seed)	100	$\exp\{-\kappa d^2\}$	1.7518828	0.0289
Original (New Seed)	70	$\exp\{-\kappa d^2\}$	1.7715113	0.03561
Original (New Seed)	40	$\exp\{-\kappa d^2\}$	1.7935544	0.04976

Table 7: Table of Posterior Means and Standard Deviation for the simulation study runs for μ_I and σ_I^2

Data-set	Total % Population Infectious	H_0	σ_I^2	
			Mean	S.D
$\alpha \times 2$	100	$(1+d^{\kappa})^{-1}$	0.9134	0.04996
$\alpha \times 2$	70	$(1+d^{\kappa})^{-1}$	0.9332	0.06434
$\alpha \times 2$	40	$(1+d^{\kappa})^{-1}$	0.8917	0.08827
$\alpha \times 2$	100	$\exp\{-\kappa d^2\}$	0.9131307	0.05003
$\alpha \times 2$	70	$\exp\{-\kappa d^2\}$	0.9321667	0.06426
$\alpha \times 2$	40	$\exp\{-\kappa d^2\}$	0.8913357	0.0879867
$\beta \times 2$	100	$(1+d^{\kappa})^{-1}$	0.9131	0.04983
$\beta \times 2$	70	$(1+d^{\kappa})^{-1}$	0.883	0.06131
$\beta \times 2$	40	$(1+d^{\kappa})^{-1}$	0.8927	0.0875
$\beta \times 2$	100	$\exp\{-\kappa d^2\}$	0.9128844	0.04977
$\beta \times 2$	70	$\exp\{-\kappa d^2\}$	0.8831906	0.06121
$\beta \times 2$	40	$\exp\{-\kappa d^2\}$	0.8928353	0.0876
$\kappa \times 2$	100	$(1+d^{\kappa})^{-1}$	0.9132	0.04992
$\kappa \times 2$	70	$(1+d^{\kappa})^{-1}$	0.9523	0.06244
$\kappa \times 2$	40	$(1+d^{\kappa})^{-1}$	0.9248	0.08094
$\kappa \times 2$	100	$\exp\{-\kappa d^2\}$	0.9130562	0.04992
$\kappa \times 2$	70	$\exp\{-\kappa d^2\}$	0.9522412	0.0625
$\kappa \times 2$	40	$\exp\{-\kappa d^2\}$	0.9250943	0.08113
Original	100	$(1+d^{\kappa})^{-1}$	0.8894	0.08347
Original	70	$(1+d^{\kappa})^{-1}$	0.9156	0.06274
Original	40	$(1+d^{\kappa})^{-1}$	0.913	0.04998
Original	100	$\exp\{-\kappa d^2\}$	0.9129414	0.04981
Original	70	$\exp\{-\kappa d^2\}$	0.9158055	0.06292
Original	40	$\exp\{-\kappa d^2\}$	0.8895275	0.08353
Original (New Seed)	100	$(1+d^{\kappa})^{-1}$	0.8338	0.04531
Original (New Seed)	70	$(1+d^{\kappa})^{-1}$	0.8385	0.05709
Original (New Seed)	40	$(1+d^{\kappa})^{-1}$	0.7834	0.07785
Original (New Seed)	100	$\exp\{-\kappa d^2\}$	0.8337618	0.04526
Original (New Seed)	70	$\exp\{-\kappa d^2\}$	0.8385418	0.05685
Original (New Seed)	40	$\exp\{-\kappa d^2\}$	0.7836434	0.0782

Table 8: Table of Posterior Means and Standard Deviation for the simulation study runs for μ_I and σ_I^2

References

[1] Anderson, T. W. & Darling, D. A. (1954) A Test of Goodness of Fit. *Journal of the American Statistical Association*, **49**, 765–769. doi:10.2307/2281537.

[2] Johnson, S. G. The Nlopt Nonlinear-optimization Package. URL: http://ab-initio.mit.edu/nlopt.

[3] Kypraios, T. (2007) *Efficient Bayesian inference for partially observed stochastic epidemics and a new class of semi-parametric time series models*. Ph.D. thesis. URL: http://www.research.lancs.ac.uk/portal/services/downloadRegister/310801/kypraios.pdf.

[4] Lau, M. S. Y., Marion, G., Streftaris, G., & Gibson, G. J. (2014) New Model Diagnostics for Spatio-temporal Systems in Epidemiology and Ecology. *Journal of The Royal Society Interface*, **11**, 20131093–20131093. arXiv:http://rsif.royalsocietypublishing.org/content/11/93/20131093.full.pdf+html, doi:10.1098/rsif.2013.1093.

[5] Nelder, J. A. & Mead, R. (1965) A Simplex Method for Function Minimization. *The Computer Journal*, **7**, 308–313. doi:10.1093/comjnl/7.4.308.

[6] Neri, F. M., Cook, A. R., Gibson, G. J., Gottwald, T. R., & Gilligan, C. A. (2014) Bayesian Analysis for Inference of an Emerging Epidemic: Citrus Canker in Urban Landscapes. *PLoS Computational Biology*, **10**, e1003587. doi:10.1371/journal.pcbi.1003587.

[7] O'Neill, P. D. (2001) Inference for an Epidemic When Susceptibility Varies. *Biostatistics*, **2**, 99–108. doi:10.1093/biostatistics/2.1.99.

[8] Parry, M., Gibson, G. J., Parnell, S., Gottwald, T. R., Irey, M. S., Gast, T. C., & Gilligan, C. A. (2014) Bayesian Inference for an Emerging Arboreal Epidemic in the Presence of Control. *Proceedings of the National Academy of Sciences*, **111**, 6258–6262. arXiv:http://www.pnas.org/content/111/17/6258.full.pdf+html, doi:10.1073/pnas.1310997111.

[9] Rowan, T. H. (1990) *Functional Stability Analysis of Numerical Algorithms*. Ph.D. thesis, University of Texas at Austin, University of Texas at Austin.
